# Robust sequential biophysical fractionation of blood plasma to study variations in the biomolecular landscape of systemically circulating extracellular vesicles across clinical conditions

**DOI:** 10.1002/jev2.12122

**Published:** 2021-08-14

**Authors:** Glenn Vergauwen, Joeri Tulkens, Cláudio Pinheiro, Francisco Avila Cobos, Sándor Dedeyne, Marie‐Angélique De Scheerder, Linos Vandekerckhove, Francis Impens, Ilkka Miinalainen, Geert Braems, Kris Gevaert, Pieter Mestdagh, Jo Vandesompele, Hannelore Denys, Olivier De Wever, An Hendrix

**Affiliations:** ^1^ Department of Human Structure and Repair Laboratory of Experimental Cancer Research Ghent University Ghent Belgium; ^2^ Cancer Research Institute Ghent Ghent Belgium; ^3^ Department of Gynecology Ghent University Hospital Ghent Belgium; ^4^ Department of Biomolecular Medicine OncoRNALab Ghent University Ghent Belgium; ^5^ Department of Internal Medicine and Pediatrics HIV Cure Research Center Ghent University Hospital Ghent Belgium; ^6^ VIB Center for Medical Biotechnology Ghent Belgium; ^7^ Department of Biomolecular Medicine Ghent University Ghent Belgium; ^8^ VIB Proteomics Core Ghent Belgium; ^9^ Biocenter Oulu University of Oulu Oulu Finland; ^10^ Department of Medical Oncology Ghent University Hospital Ghent Belgium

**Keywords:** biomarkers, blood, corona, exosomes, extracellular vesicles, isolation, lipoprotein particles, proteomics, separation, transcriptomics

## Abstract

Separating extracellular vesicles (EV) from blood plasma is challenging and complicates their biological understanding and biomarker development. In this study, we fractionate blood plasma by combining size‐exclusion chromatography (SEC) and OptiPrep density gradient centrifugation to study clinical context‐dependent and time‐dependent variations in the biomolecular landscape of systemically circulating EV. Using pooled blood plasma samples from breast cancer patients, we first demonstrate the technical repeatability of blood plasma fractionation. Using serial blood plasma samples from HIV and ovarian cancer patients (n = 10) we next show that EV carry a clinical context‐dependent and/or time‐dependent protein and small RNA composition, including miRNA and tRNA. In addition, differential analysis of blood plasma fractions provides a catalogue of putative proteins not associated with systemically circulating EV. In conclusion, the implementation of blood plasma fractionation allows to advance the biological understanding and biomarker development of systemically circulating EV.

## 1 INTRODUCTION

In addition to cells and platelets, blood contains a diversity of lipid carrying particles including extracellular vesicles (EV) and lipoprotein particles (LPP), as well as small and large molecular weight proteins (Simonsen, 2017; Tulkens et al., 2020a). EV are nanometer‐sized membrane particles composed of different lipids (especially phospholipids and cholesterol), soluble and transmembrane proteins, metabolites and nucleic acids. EV are released by most cells of the body, also under pathological conditions such as cancer, through orchestrated plasma membrane budding generating microvesicles or fusion of multi‐vesicular endosomes with the plasma membrane releasing exosomes. A third mechanism of EV genesis takes place during controlled cell death when cells fragment into apoptotic bodies (Van Niel et al., 2018). The analysis of EV as biomarkers to detect and monitor disease has gained widespread interest, mainly because they are accessible in body fluids and carry a vast variety of biological cargos reflecting the pathophysiological processes occurring within the parent cell, offering perspectives for multi‐analyte biomarker measurements (De Wever & Hendrix, 2019; Dhondt et al., 2020; Kalluri & Lebleu, 2020). Chylomicrons (CM), very‐low‐density lipoproteins (VLDL), intermediate density lipoproteins (IDL), low‐density lipoproteins (LDL), and high‐density lipoproteins (HDL) are LPP mainly responsible for systemic lipid transport. In addition, LPP carry proteins and nucleic acids (small RNA in particular) (Allen et al., 2018; Dashty et al., 2014; Shah et al., 2013) with biomarker potential (Burillo et al., 2016; Emmens et al., 2018). Compared to LPP, EV are a minor population with an estimated 6 orders of magnitude lower concentration (approximately 10^10^ EV per ml) in blood plasma (Geeurickx et al., 2019; Johnsen et al., 2019). The concentration range of low and high molecular weight proteins extends 12–13 orders of magnitude, with  >  90% of all plasma protein content covered by a few highly abundant proteins. These are primarily haemostatic (e.g., prothrombin), acute phase response proteins (e.g., serpins), protein transporters, immunoglobulins and the colloid osmotic pressure protein albumin (Geyer et al., 2017).

Separating EV, LPP and highly abundant plasma proteins remains challenging and complicates the biological understanding and biomarker development of EV (De Wever & Hendrix, 2019; Dhondt et al., 2020; Hendrix, 2021). EV and LPP have been separated from blood plasma using a wide diversity of methods including differential ultracentrifugation, size‐exclusion chromatography and density gradient centrifugation (Coumans et al., 2017; Kuklenyik et al., 2018; Van Deun et al., 2017). Given that EV and LPP overlap, at least in part, in size and density, the sequential use of methods is inevitable to increase specificity (also referred to as purity) of EV preparations (Simonsen, 2017; De Wever & Hendrix, 2019). We previously reported the sequential implementation of size‐exclusion chromatography and density gradient centrifugation to discover systemically circulating EV of bacterial origin (bacterial EV or BEV), underpinning the suitability of this approach for biomarker development (Tulkens et al., 2020b). Variations on this protocol have been described for the study of EV in blood plasma, including the use of a density cushion instead of a density gradient or a density gradient followed by bind‐elute chromatography (Karimi et al., 2018; Onódi et al., 2018; Zhang et al., 2020).

In this study, we evaluate and implement sequential biophysical fractionation of blood plasma to analyse clinical context‐dependent and time‐dependent variations in the biomolecular landscape of EV with high repeatability, with the aim to advance the biological understanding and biomarker development of systemically circulating EV.

## 2 MATERIALS AND METHODS

### 2.1 Blood samples

Venous blood was collected using Venosafe‐citrate tubes (VF‐054SBCS07, Terumo Europe, Leuven, Belgium) from healthy donors, breast and ovarian cancer patients, and HIV patients (Figure S1). Blood samples were collected from donors providing informed consent according to the Ethical Committee of Ghent University Hospital approval EC/2014/0655 and in accordance to relevant guidelines. All blood samples were collected, stored and handled by the Laboratory of Experimental Cancer Research (Ghent University, Belgium). All blood samples were first characterized (complete blood count) using the hematology analyzer (XP‐300, Sysmex, Hoeilaart, Belgium). Platelet‐depleted blood plasma was prepared by two serial centrifugations at 2500 g for 15 min at room temperature. Platelet‐depletion was confirmed using the hematology analyzer. Absence of haemolysis was confirmed by the lack of spectrophotometric absorbance peak of free haemoglobin at 414 nm using a BioDrop DUO spectrophotometer (BioDrop, Cambridge, United Kingdom). All blood samples were processed within 120 min after blood collection and platelet‐depleted blood plasma was stored as 1 ml aliquots at ‐80°C. A pool of healthy donor blood and breast cancer patient blood was collected and prepared to evaluate the technical repeatability of the sequential biophysical fractionation strategy (Figure S1). Serial blood samples were collected and prepared from ovarian cancer patients and HIV patients to evaluate time‐dependent variations in the biomolecular landscape of EV (Figure S1). Healthy donors, breast cancer patients and HIV patients were non‐fasting at the time of blood sampling. Ovarian cancer patients, which were serially sampled, were all fasting at time‐point 1 (blood sampling prior to surgery) and two ovarian cancer patients were fasting at time point 5; other samples were obtained from non‐fasting patients.

### 2.2 Size‐exclusion chromatography

Crude extracts from blood plasma were prepared using size‐exclusion chromatography columns with Sepharose CL‐2B as previously described (Tulkens et al., 2020a). Sepharose CL‐2B (GE Healthcare, Uppsala, Sweden) was washed 3 times with elution buffer (PBS pH 7.2 containing 0.32% trisodium citrate dihydrate) (ChemCruz, Dallas, Texas, USA). A SEC column was prepared by placing a nylon net with 20 μm pore size (NY2002500, Merck Millipore, Billerica, Massachusetts, USA) on the bottom of a 10 ml syringe (BD Biosciences, San Jose, California, USA), followed by stacking of 10 ml Sepharose CL‐2B. On top of one SEC column, 2 ml blood plasma was loaded followed by elution and collection of 16 sequential 1 ml eluate fractions. In general, 6 ml of blood plasma was fractionated using three parallel SEC columns; corresponding fractions of the three columns were pooled and concentrated to 1 ml using a 10 kDa centrifugal filter (Amicon Ultra‐2 ml, Merck Millipore, Billerica, Massachusetts, USA) (Vergauwen et al., 2017). SEC fractions 5 and 6, identified as EV‐containing fractions and referred to as the crude extract, were pooled, concentrated to 1 ml using a 10 kDa centrifugal filter and loaded on top of an OptiPrep density gradient (ODG). Alternatively, we implemented qEV columns according to manufacturer's protocol (Izon). Briefly, qEV columns were equilibrated using 10 ml PBS buffer. On top of the qEV column, 1 ml blood plasma was loaded followed by elution and collection of 15 sequential 500 μl eluate fractions.

### 2.3 OptiPrep density gradient centrifugation

OptiPrep (Axis‐Shield, Oslo, Norway) density gradients were prepared as previously described (Tulkens et al., 2020a; Van Deun et al., 2014). Briefly, a discontinuous iodixanol gradient was prepared by layering 4 ml of 40%, 4 ml of 20%, 4 ml of 10% and 3.5 ml of 5% iodixanol in a 16.8 ml open top polyallomer tube (Beckman Coulter, Fullerton, California, USA). One millilitre of crude extract was placed on top of the gradient, followed by 18 h ultracentrifugation at 100 000 g and 4°C using a SW 32.1 Ti rotor (Beckman Coulter, Fullerton, California, USA). Fractions of 1 ml were collected and ODG fractions corresponding to a density of 1.04‐1.07 g/ml were pooled, as well as ODG fractions corresponding to a density of 1.09‐1.10 g/ml. SEC was performed on these pooled ODG fractions (identical to the first SEC) to remove iodixanol (Tulkens et al., 2020a; Vergauwen et al., 2017). SEC fractions 4–7 were pooled, concentrated to 100 μl, stored at ‐80°C until further use and referred to as the LPP extract (1.04‐1.07 g/ml) and EV extract (1.09‐1.10 g/ml).

### 2.4 Protein measurements

The protein concentration of samples was measured using the Qubit fluorometer 3.0 (ThermoFisher, Waltham, Massachusetts, USA), according to the manufacturer's instructions. EV protein concentration was measured after lysis with 0.4% SDS. Sample preparation was done by a 1:1 dilution with Laemmli 1x lysis buffer (0.125 M Tris‐HCl (pH 6.8), 10% glycerol, 2.3% sodium dodecyl sulfate (SDS)). Analysis was performed with a BioDrop DUO spectrophotometer (BioDrop, Cambridge, United Kingdom). Particle enrichment was calculated by the ratio of particle count (nanoparticle tracking analysis) to protein, APOA1‐containing and APOB‐containing particles in each fraction compared to the same ratio in total blood plasma.

### 2.5 Antibodies

The following antibodies were used for immunostaining: mouse monoclonal anti‐APOA1 (1:100, B10, Santa Cruz Biotechnology, Dallas, Texas, USA), anti‐Flot‐1 (1:1000, 610820, BD Biosciences, Franklin Lakes, New Jersey, USA), sheep anti‐mouse horseradish peroxidase‐linked antibody (1:4000, NA931V, Cytiva, Massachusetts, USA) and donkey anti‐rabbit horseradish peroxidase‐linked antibody (1:3000, NA934V, Cytiva, Massachusetts, USA).

### 2.6 Enzyme‐linked immunosorbent assay (ELISA) and TRIFic assay

APOA1, APOB and CD9 quantification were performed using the Human Apolipoprotein A‐I Quantikine ELISA Kit (DAPA10, R&D Systems, Minneapolis, USA), Apolipoprotein B (APOB) Human SimpleStep ELISA Kit (ab190806, Abcam, Cambridge, UK) and the TRIFic CD9 exosome assay (EX101, Cell Guidance Systems, Carlsbad, California, USA) according to the manufacturer's protocol respectively. Data measurements were obtained using the BioTek Synergy HTX S1LFA and Gen5 software (BioSPX, Drogenbos, Belgium) and the Paradigm Detection Platform (Beckman Coulter, Fullerton, California, USA) and SoftMax Pro 6.1 software (Molecular Devices, Sunnyvale, California, USA).

### 2.7 SDS‐PAGE and Western blotting

Samples were dissolved in reducing sample buffer (0.5 M Tris‐HCl (pH 6.8), 40% glycerol, 9.2% SDS, 3% 2‐mercaptoethanol, 0.005% bromophenol blue) and heated at 95°C during 5 min. Proteins were separated by SDS‐polyacrylamide gel electrophoresis and transferred to nitrocellulose membranes (Bio‐Rad, Hercules, California, USA). After blocking the membranes with 5% non‐fat milk in PBS with 0.5% Tween 20, the blots were incubated overnight with primary antibodies. Incubation with secondary antibodies was performed after extensive washing of the membranes in PBS with 0.5% Tween 20. After final extensive washing, chemiluminescence substrate (WesternBright Sirius, Advansta, Menlo Park, California, USA) was added and imaging was performed using the Proxima 2850 Imager (IsoGen Life Sciences, De Meern, The Netherlands). When indicated, an proteinase K (PK) (P6556‐100MG, Sigma, Diegem, Belgium) treatment was performed. This was done at a PK concentration of 1 mg/ml for 60 min at 37 °C and was ended at 4 °C.

### 2.8 Coomassie brilliant blue staining

Equal volumes of all SEC fractions were dissolved in reducing sample buffer (1 M Tris‐HCl (pH 6.8), 30% glycerol, 6% SDS, 3% 2‐mercaptoethanol, 0.005% bromophenol blue) and heated at 95°C during 5 min. Proteins were separated by SDS‐polyacrylamide gel electrophoresis. Gel was stained with Coomassie Brilliant Blue R‐250 (Bio‐Rad, Hercules, California, USA) during 1 h followed by destaining overnight at 4°C with Coomassie Brilliant Blue destaining solution (Bio‐Rad).

### 2.9 Nanoparticle tracking analysis

Size distributions of samples were generated by nanoparticle tracking analysis (NTA) using a NanoSight LM10 microscope (NanoSight, Amesbury, UK) equipped with 405 nm laser. For each analysis, three videos of 60 s were recorded and analysed with camera level 13 and detection threshold 3. The temperature was monitored during recording. Recorded videos were analysed with the NTA Software version 3.0. For optimal measurements, samples were diluted with PBS until particle concentration was within the concentration range for the NTA Software (3 × 10^8^–10^9^ particles/ml).

### 2.10 Transmission electron microscopy

The composition of crude, LPP and EV extracts was analysed by transmission electron microscopy (TEM). Samples were deposited on Formvar carbon‐coated, glow discharged grids, which were stained with uranylacetate and embedded in methylcellulose/uranylacetate. Prepared grids were then examined using a Tecnai Spirit transmission electron microscope (FEI, Eindhoven, The Netherlands) and images were captured with Quemasa charge‐coupled device camera (Olympus Soft Imaging Solutions GMBH, Munster, Germany).

### 2.11 LC‐MS/MS analysis

Starting from 6 ml blood plasma, 50% of the obtained volumes of crude, LPP and EV extracts were first reduced by vacuum drying to 1.5 ml. Amphipol A8‐35 (CliniSciences, Nanterre, France) at 1 mg/ml was added to the samples, vortexed and incubated for 10 min at room temperature (pH 7). Next, the samples were reduced and alkylated with respectively 15 mM TCEP and 30 mM iodoacetamide for 15 min in the dark at 37 °C. The samples were acidified with 5% formic acid to pH 3 and centrifuged for 10 min at 16,000  ×  g. The resulting protein containing pellets were re‐dissolved in 0.5 ml 50 mM ammonium bicarbonate pH 8.0 followed by overnight digestion with 2.5 μg trypsin at 37 °C. Following trypsin digestion, the samples were acidified to pH 3 resulting in Amphipol A8‐35 precipitation. The samples were centrifuged for 10 min at 16,000  ×  g at room temperature to pellet precipitated Amphipol A8‐35, while peptides remain in solution. The supernatant containing the peptide material was concentrated by vacuum drying to 20 μl of which 8 μl was injected for LC‐MS/MS analysis on an Ultimate 3000 RSLCnano system (Thermo Fischer scientific, Erembodegem, Belgium) in‐line connected to a Q Exactive mass spectrometer (Thermo Fischer scientific, Erembodegem, Belgium). Trapping was performed at 10 μl/min for 4 min in trapping solvent (0.1% TFA in water/acetonitrile (98:2, v/v)) on a 100 μm internal diameter (I.D.)  ×  20 mm trapping column (5 μm beads, C18 Reprosil‐HD, Dr. Maisch, Germany) and the sample was loaded on a reverse‐phase column (made in‐house, 75 μm I.D. x 150 mm, 3 μm beads C18 Reprosil‐HD, Dr. Maisch). Peptides were eluted by a linear increase from 2 to 55% solvent B (0.1% formic acid in water/acetonitrile (2:8, v/v)) over 120 min at a constant flow rate of 300 nl/min. The mass spectrometer was operated in data‐dependent mode, automatically switching between MS and MS/MS acquisition for the 10 most abundant ion peaks per MS spectrum. Full‐scan MS spectra (400–2000 m/z) were acquired at a resolution of 70,000 in the orbitrap analyzer after accumulation to a target value of 3,000,000. The 10 most intense ions above a threshold value of 17,000 were isolated (window of 2.0 Th) for fragmentation at a normalized collision energy of 25% after filling the trap at a target value of 50,000 for maximum 60 ms. MS/MS spectra (200–2000 m/z) were acquired at a resolution of 17,500 in the orbitrap analyzer. The S‐lens RF level was set at 50 and we excluded precursor ions with single, unassigned and charge states above 5 from fragmentation selection.

Data analysis was performed with MaxQuant (version 1.5.4.1) using the Andromeda search engine with default search settings including a false discovery rate set at 1% on both the peptide and protein level. Spectra were searched against the human protein entries in the Swiss‐Prot database (downloaded from http://www.uniprot.org, version from May 2016 containing 20,195 human protein sequences). The mass tolerance for precursor and fragment ions were set to 4.5 and 20 ppm, respectively, during the main search. Enzyme specificity was set as C‐terminal to arginine and lysine, also allowing cleavage at proline bonds with a maximum of two missed cleavages. Carbamidomethylation of cysteine residues was set as fixed modification. Variable modifications were set to oxidation of methionine residues and acetylation of protein N‐termini. Only proteins with at least one unique or razor peptide were retained. Proteins were quantified by the MaxLFQ algorithm integrated in the MaxQuant software. All raw data were submitted to the PRIDE database (PXD023001).

### 2.12 Proteomic data analysis

Identified protein lists were analysed and visualized using the Perseus software version 1.5.4.1 (Tyanova et al., 2016). Reverse database hits and contaminant proteins were removed and LFQ protein intensities were log2 transformed. Proteins showing valid values in at least 66.67% of at least one group were selected. Missing values were imputed from the observed normal distribution of intensities. For selected analyses, intensities were transformed to z‐scores. Differences of the mean were evaluated by Student's t‐test with permutation‐based false discovery rate (FDR) estimation and q‐values smaller than 0.05 were considered statistically significant. Volcano plots were generated using the Perseus software with S_0_ set at 0.1. Principle component analyses were performed using Past3 software (Hammer et al., 2001). Unsupervised hierarchical clustering heat maps, using 1‐Pearson correlation, were generated using the Morpheus tool. Protein annotation and gene ontology analysis was implemented using UniProt, GeneCards and the DAVID database. Functional pathway enrichment analysis was performed using the ClueGo plug‐in (Bindea et al., 2009) and data were visualized using Cytoscape (Shannon, 2003). Identification of LDL and HDL‐associated proteins was obtained using the putative LDL/HDL proteome database from the Davidson/Shah lab (Davidson).

### 2.13 Small RNA sequencing

Starting from 6 ml blood plasma, 50% of the obtained volumes of EV extracts were used for RNA extraction using the miRNeasy serum/plasma kit (Qiagen, Hilden, Germany). In case of total plasma, RNA was isolated from 200 μl platelet‐depleted plasma. Of the 12 μl eluate, 5 μl was used as input for the small RNA library preparation protocol. Sequence libraries were generated using the TruSeq small RNA library prep (Illumina, San Diego, California, USA) following manufacturer's instructions with small modifications. After PCR amplification, quality of libraries was assessed using a high sensitivity DNA kit on a Bioanalyzer (Agilent, Santa Clara, California, USA) according to manufacturer's instructions. Size selection was performed using 3% agarose dye‐free marker H cassettes on a Pippin Prep (Sage Science, Beverly, Massachusetts, USA) following manufacturer's instructions with a specified collection size range of 125–153 bp. Libraries were further purified and concentrated by ethanol precipitation, resuspended in 10 μl of 10 mM tris‐HCl (pH  =  8.5) and quantified using qPCR. Based on KAPA qPCR, equimolar library pools were prepared, quality was assessed (as described above) and the library was further diluted to 4 nM using 10 mM tris‐HCl (pH  =  8.5). The pooled library was then sequenced at a final concentration of 1.2 pM on a NextSeq 500 (Illumina, San Diego, California, USA) using a high output v2 kit (single‐end, 75 cycles, Illumina). All raw data were submitted to the BioProject database (PRJNA685637).

### 2.14 Sequencing data analysis

For the quantification of small RNA, Biogazelle's dedicated small RNA‐seq pipeline was used (part of their Cobra suite). As publicly available alternatives, we advise miRDeep2 and miRExpress. In short, adaptor trimming was performed using Cutadapt v1.8.1: reads shorter than 15 bp and those in which no adaptor was found, were discarded. For quality control, the FASTX‐Toolkit (v0.0.14) was used, a minimum quality score of 20 in at least 80% of bases was applied as a cutoff. The reads were mapped with Bowtie (v1.1.2) without allowing for mismatches. Mapped reads were annotated by matching genomic coordinates of each read with genomic locations of miRNAs (obtained from miRbase, v20) and other small RNA (obtained from UCSC (human: GRCh37/hg19) and Ensembl, v84). Genomic features with a zero read count across all samples and without read count ≥ 1 in at least half of the samples of one group were removed. Additionally, reads were also uploaded to the Genboree Workbench and analysed using the exceRpt small RNA‐seq pipeline (exceRptPipeline_v4.6.2). The selected endogenous genome was hg19. For non‐ribosomal alignment we used following databases (ordered by priority): miRbase (v21), gtRNAdb, piRNABank, Gencode (v18) and circBase. Reads shorter than 18 nt were discarded and a minimum quality score of 20 in at least 80% of bases was applied as a cutoff. One mismatch was allowed.

Normalization of read counts, log2 transformation and differential expression analyses were performed using the R statistical programming language (version 3.4.0) and the R package DESeq2 (v1.16.1).

Hierarchical clustering was performed using Manhattan distance and the Ward's method. Criteria for differential gene expression were set at log2 fold‐change ≥1 and an adjusted (Benjamini‐Hochberg) p‐value ≤ 0.05. Correlations in RNA sequencing data were based on Spearman correlation.

Gene Set Enrichment Analysis (GSEA) (Java version 2‐2.2.3) (Subramanian et al., 2005) was performed using two gene lists representing the top 50 miRNA from platelets and HDL (Plé et al., 2012; Vickers et al., 2011) (Figure S7). Small RNAs were ranked based on log2 fold‐change values from the differential gene expression analysis between total blood plasma and EV extracts from the breast cancer patient blood plasma pool. We selected 1000 permutations and ‘classic’ enrichment score as input parameters.

The Database for Annotation, Visualization and Integrated Discovery (DAVID, version 6.8) was used for KEGG pathway analysis and gene ontology.

### 2.15 Statistical analysis

Data analysis and graphical presentations were performed using GraphPad Prism version 7 (GraphPad Software, San Diego, California, USA). The body of the box plots represents the first and third quartiles of the distribution, and the median line. The whiskers comprise the minimum and maximum values. Kernel density plots were obtained using online statistical tool (http://www.wessa.net/rwasp_density.wasp). Statistical calculations were performed using MedCalc (version 11.0; MedCalc Software). Mann‐Whitney U tests were performed to compare non‐normally distributed continuous variables. Pearson r correlation was calculated to measure the degree of relationship between linearly related variables. Spearman rank correlation was calculated to measure the degree of association between two variables. P values smaller than 0.05 were considered to be statistically significant (*P < 0.05, ** *P* < 0.01). Data in text are represented by mean/median value ± standard error. Illustrations were made in Adobe Illustrator CS6.

### 2.16 EV‐TRACK

We have submitted all relevant data of our experiments to the EV‐TRACK knowledgebase (EV‐TRACK ID: EV200081) (Van Deun et al., 2017).

## 3 RESULTS

### 3.1 Sequential biophysical fractionation of blood plasma in crude, EV and LPP extracts

**FIGURE 1:**
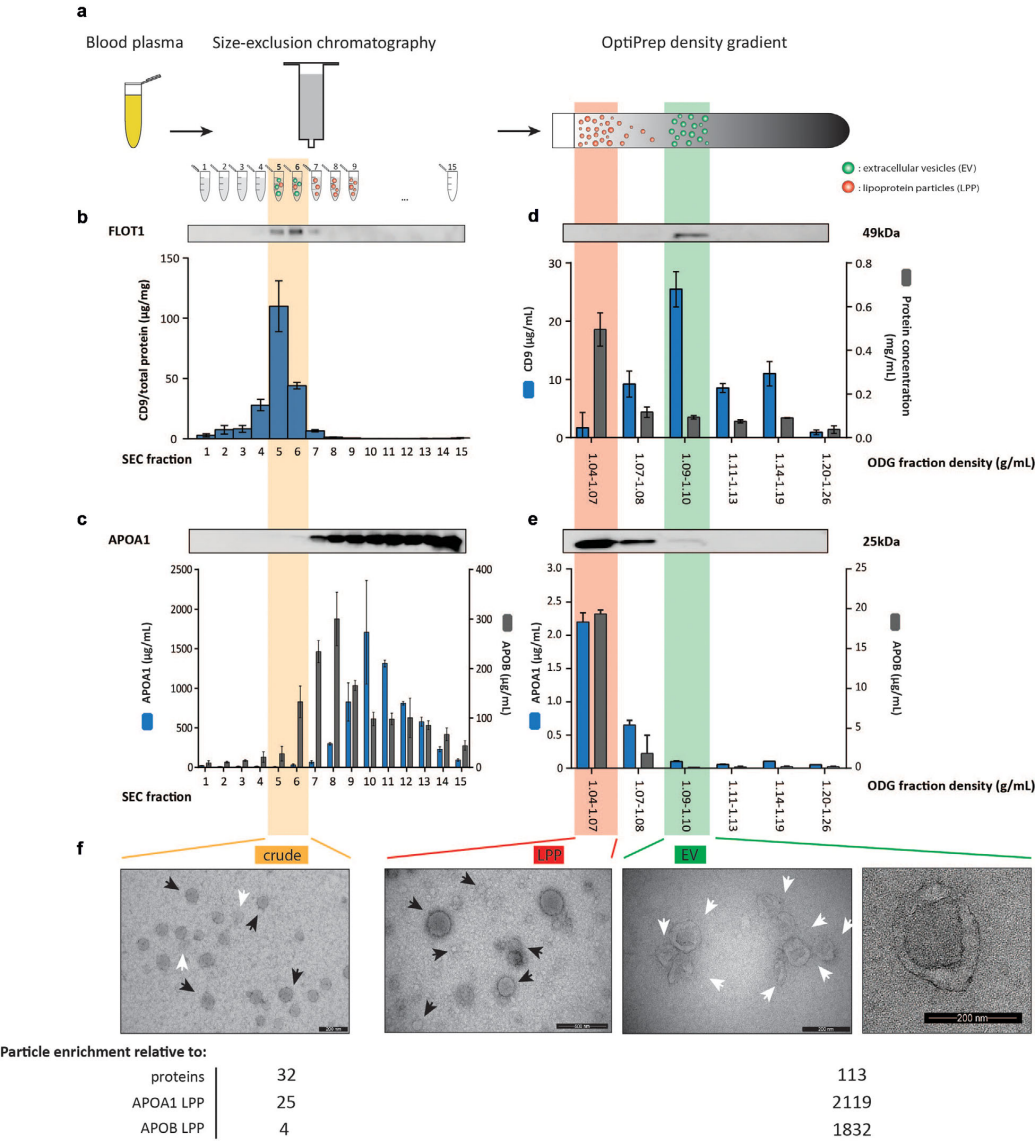
Characterization of crude, LPP and EV extracts obtained by size and density‐based fractionation of blood plasma. (a) Graphical overview of the sequential biophysical separation approach including size‐exclusion chromatography (SEC) and OptiPrep density gradient (ODG) centrifugation. (b) Western blot (FLOT1) and ELISA analysis (CD9) of SEC fractions. (c) Western blot (APOA1) and ELISA analysis (APOA1, APOB) of SEC fractions. (d) Western blot (FLOT1), ELISA analysis (CD9) and protein concentration of ODG fractions. (e) Western blot (APOA1) and ELISA analysis (APOA1, APOB) of ODG fractions. (f) TEM analysis of crude (scale bar: 200 nm), LPP (scale bar: 500 nm) and EV extracts (scale bar: 200 nm (left and right)) and overview of the particle enrichment relative to proteins, and APOA1‐containing and APOB‐containing LPP. All SEC and ODG fractions were loaded in equal volumes for western blot analysis. Note the differential axis labelling in (b) (expressing CD9 in μg/mg) and (d) (expressing CD9 in μg/ml)

We integrated size‐exclusion chromatography (SEC) followed by OptiPrep density gradient (ODG) centrifugation to fractionate blood plasma in two dimensions (size and density) (Figure 1a) (Tulkens et al., 2020a). Healthy donor blood plasma (2 ml) was loaded on a SEC column as previously described (Tulkens et al., 2020a, 2020b; Vergauwen et al., 2017). Coomassie Brilliant Blue analysis and protein concentration measurements of eluted SEC fractions 1 to 15 revealed a Gaussian protein elution profile from SEC fraction 5 onwards (Figure S2, A and B). ELISA identified 73.0% of the total EV‐associated tetraspanin CD9 in SEC fractions 5 and 6 (Figure 1b). Western blot analysis for luminal membrane‐associated FLOT1 confirmed the enrichment of EV in SEC fractions 5 and 6 (Figure 1b). The combination of SEC fractions 5 and 6 recovered 34% of particles (quantified by NTA) and excluded 98.9% of proteins from the total sample elute (quantified by protein concentration measurements) (Figure S2, B and C) resulting in a 32‐fold enrichment of particles compared to protein (Figure S2D). Densitometry of the albumin bands after Coomassie Brilliant Blue staining confirmed that only 0.078% of total albumin was retained in SEC fractions 5 and 6. Western blot analysis and ELISA revealed that the bulk of APOA1, the primary lipoprotein of HDL particles, eluted from SEC fraction 7 onwards. SEC fractions 5 and 6 retained 1.40% of total lipoprotein APOA1 (Figure 1c), resulting in a 25‐fold enrichment of total particles (quantified by NTA) relative to APOA1‐containing LPP (quantified by ELISA) (Figure S2D). This minimal co‐elution of EV and APOA1 was also observed using commercially available SEC columns and was not prevented by a four‐fold reduction in blood plasma sample loading volume (Figure S2, E and F). ELISA for APOB, the primary lipoprotein of LDL, IDL, VLDL and CM particles revealed that SEC fractions 5 and 6 contained 9.67% of total lipoprotein APOB, resulting in a 4‐fold enrichment of total particles (quantified by NTA) relative to APOB‐containing LPP (quantified by ELISA) (Figure 1c). TEM confirmed the presence of substantial numbers of LPP in SEC fractions 5 and 6 (Figure 1f). Hereafter we refer to SEC fractions 5 and 6 as “crude extract”, representing a sample that predominantly contains lipid‐carrying particles including EV, and APOA1 and APOB‐containing LPP particles, but is depleted of abundant plasma proteins.

Next, the crude extract was submitted to density‐based separation. SEC fractions 5 and 6 were pooled, concentrated by ultrafiltration to 1 ml and loaded on top of an OptiPrep density gradient. FLOT1 and CD9 were enriched at higher density fractions (1.09‐1.10 g/ml) (Figure 1d). ELISA identified the highest signal of CD9 in this density fraction, equal to 44.6% of the total CD9 signal in all density fractions (Figure 1d). TEM confirmed the enrichment of EV, visualized as double membrane structures varying between 50 and 250 nm in size (Figure 1f), in these fractions. Hereafter we refer to ODG fraction 1.09‐1.10 g/ml as “EV extract”, representing a sample derived from the crude extract that is further enriched for EV while depleted of LPP. To validate the EV extract as an EV‐enriched fraction, we spiked 10^10^ Green Fluorescent Protein (GFP)‐positive EV (quantified by NTA) in healthy donor plasma followed by sequential SEC and ODG centrifugation. Western blot analysis confirmed the presence of GFP‐positive EV in both crude and EV extracts (Figure S3 and Supplementary Materials & Methods). More than 85% of APOA1 and more than 95% of APOB from the crude extract was retained in the lower density fractions of the gradient (1.04‐1.07 g/ml) containing the highest protein concentration (Fig. 1d and e). TEM further confirmed the enrichment of LPP in these fractions (Figure 1f). Hereafter we refer to ODG fraction 1.04‐1.07 g/ml as “LPP extract”, representing a sample derived from the crude extract that is further enriched for LPP while depleted of EV. We calculated that the EV extract recovered 0.685% of total plasma particles (quantified by NTA), 0.00607% of plasma protein (quantified by protein concentration measurements), 0.000323% of plasma APOA1 and 0.0004% of plasma APOB (quantified by ELISA), resulting in a 113‐fold enrichment of particles compared to protein, a 2119‐fold relative depletion of APOA1‐containing LPP and a more than 1800‐fold relative depletion of APOB‐containing LPP (Figure 1f and Fig. S2D).

### 3.2 Identification of the differential proteome landscape of crude, EV and LPP extracts with high repeatability

Technical replicates (n = 6) derived from a pool of blood plasma collected from breast cancer patients (Figure S1), to discard inter‐patient variations, were subjected to sequential SEC and ODG, and crude, EV and LPP extracts were analysed by mass spectrometry‐based proteomics (LC‐MS/MS).

**FIGURE 2:**
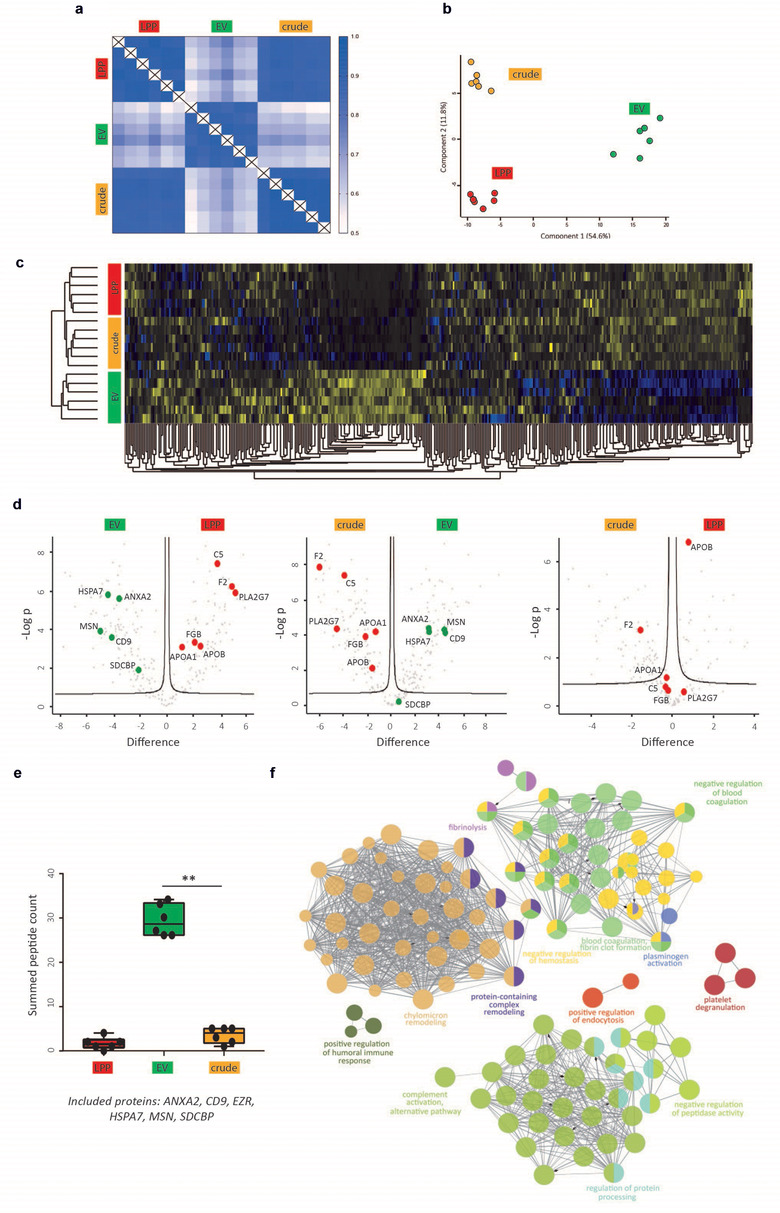
Differential analysis of the protein landscape of crude, LPP and EV extracts. (a) Correlation matrix of LFQ protein intensities, (b) PCA and (c) unsupervised hierarchical clustering and heatmap analysis of the protein composition of crude, LPP and EV extracts (n = 6 technical replicates). (d) Volcano plot analysis revealing the differential expression of EV and non‐EV‐associated proteins in crude, LPP and EV extracts. (e) Summed peptide count of EV‐associated proteins (ANXA2, CD9, EZR, HSPA7, MSN and SDCBP) in crude, LPP and EV extracts (Mann‐Whitney U test, *P* = 0.0022). (f) Functional pathway analysis of the selected group of 83 putative non‐EV associated proteins

Correlation analysis of LFQ protein intensities revealed that the technical repeatability within a certain extract type was high with median Pearson correlation coefficients for crude extracts of 0.916 ± 0.0221; for LPP extracts of 0.905 ± 0.181; and for EV extracts of 0.877 ± 0.0186 (Figure 2a). This analysis further revealed that the correlation between crude and EV extracts, and LPP and EV extracts was low with median Pearson's r coefficients down to 0.624 ± 0.0509; and 0.642 ± 0.0561, respectively. Interestingly, correlation between crude and LPP extracts was high with a median correlation coefficient of 0.865 ± 0.0112.

To further evaluate variations in the protein composition of the crude, LPP and EV extracts, unsupervised hierarchical clustering and principal component analysis (PCA) were performed. Both showed differential clustering of EV extracts from LPP and crude extracts, indicative of a disparate protein landscape in EV extracts, (Figure 2b (PC 1: 54.6% of the variation) and Figure 2c). Kernel density plots, representing the distribution of protein differences, identified a Gaussian distribution for crude versus LPP extracts further confirming low biological variability in protein composition between both sample groups. In contrast, Kernel density plots of both crude versus EV extracts and LPP versus EV extracts showed a bimodal distribution supporting the conclusion from the heatmap and PCA that EV extracts differ in protein composition from LPP and crude extracts (Figure S4).

Volcano plot analysis comparing EV extracts with LPP and crude extracts revealed differentially enriched proteins between the sample groups (Figure 2d). EV‐associated proteins (ANXA2, CD9, HSPA7, MSN and SDCBP) were significantly enriched in EV extracts (Figure 2d and Fig. S5A). The summed peptide count of a set of EV‐associated proteins was significantly reduced in crude and LPP extracts compared to EV extracts (Figure 2e, *P* = 0.0022, Mann‐Whitney U test). In contrast, C5, F2, PLA2G7, FGB, APOA1 and APOB were significantly enriched in crude and LPP extracts (Figure 2d and Figure S5A). None of the EV‐associated proteins were detected in more than 66% of crude and LPP extracts and are therefore not present in the volcano plot on the right (Figure 2d). Previously reported putative HDL‐associated proteins (Davidson) made up 41.3% and 55.1% of the total proteins enriched in the crude and LPP extracts, respectively, while this was only 10.3% in EV extracts (Figure S5B). Additionally, EV extracts also contained the lowest relative proportion of putative LDL‐associated proteins compared to crude and LPP extracts. Functional pathway analysis revealed enrichment for signalling and immunity‐related pathways in EV extracts, while complement and platelet pathways were enriched in crude extracts (Figure S5C).

Next, we performed an in‐depth comparative analysis of crude, LPP and EV extracts to identify a group of putative contaminants of EV extracts. Hereto, we took into account two selection criteria resulting in 83 putative contaminating proteins (Table S1): (1) proteins have a similar retention time during SEC and thus are identified in the crude extract, and (2) proteins have a different density compared to EV and thus are significantly enriched (Student's t‐test corrected for multiple testing, *P* < 0.05) in LPP extracts. Functional pathway analysis of these putative contaminating proteins revealed the enrichment of non‐EV associated pathways, such as chylomicron remodelling, blood coagulation and fibrin clot formation, and complement activation (Figure 2f).

Taken together, these results indicate that the sequential biophysical separation approach has a high technical repeatability, enriches EV‐associated proteins in EV extracts while depleting LPP‐associated proteins, that are retained in LPP extracts, and supports the identification of putative non‐EV associated proteins by differential analysis of crude, LPP and EV extracts.

### 3.3 Context‐dependent analysis of the proteome landscape of EV and LPP extracts across clinical conditions

Blood plasma samples collected from breast cancer patients (pooled, 6 technical replicates) , ovarian cancer patients (4 patients, 5 time points) and HIV patients (6 patients, 2 time points) (described in Figure S1) were subjected to sequential SEC and ODG, and EV and LPP extracts were analysed by mass spectrometry‐based proteomics (LC‐MS/MS).

**FIGURE 3:**
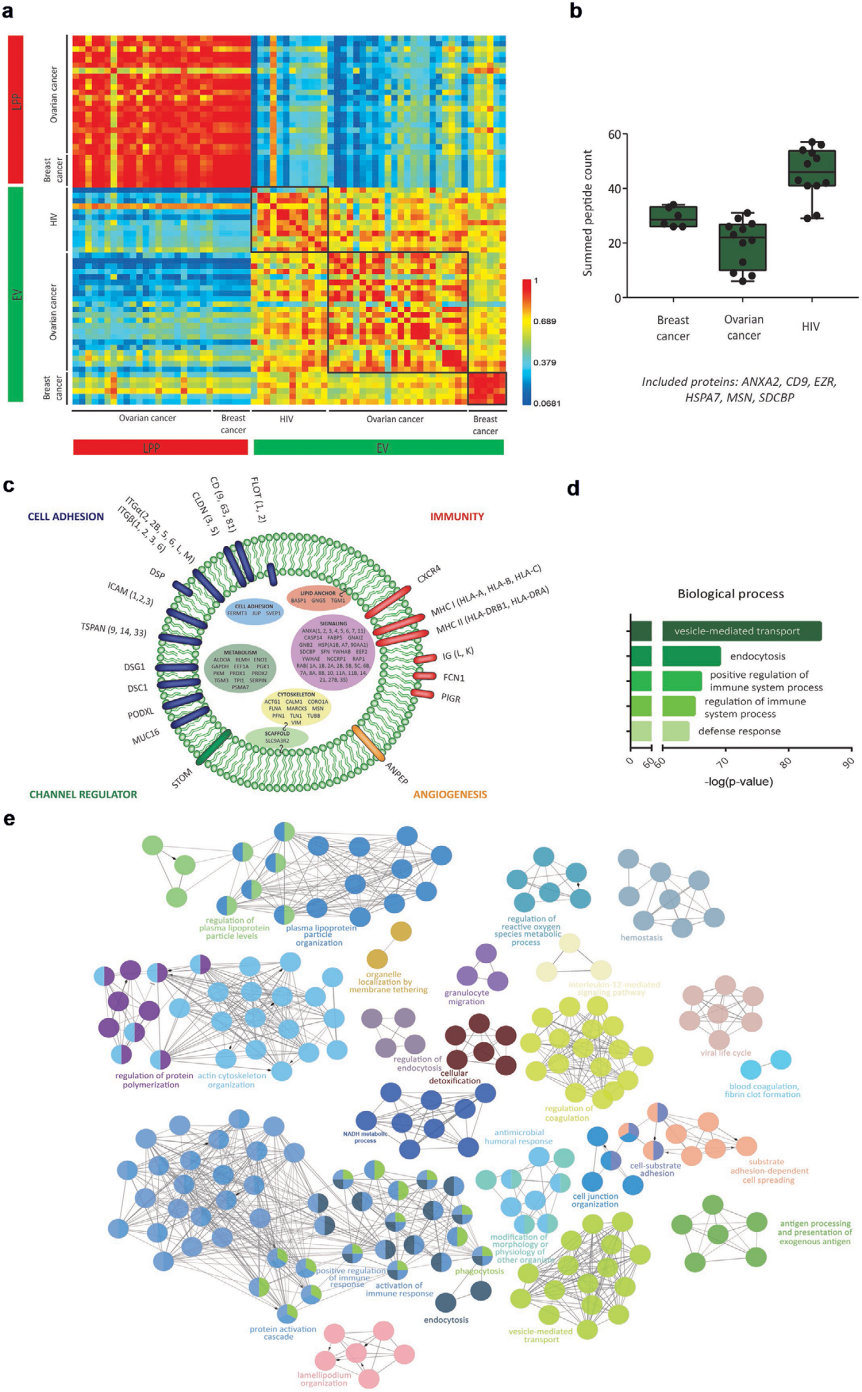
Clinical context‐dependent analysis of protein landscape of EV and LPP extracts. (a) Correlation matrix of LFQ protein intensities of EV and LPP extracts obtained by fractionation of blood plasma from breast cancer patients (n = 6), ovarian cancer patients (n = 4; 5 time points) and HIV patients (n = 6; 2 time points). (b) Summed peptide count of EV‐associated proteins (ANXA2, CD9, EZR, HSPA7, MSN and SDCBP) in EV extracts across clinical conditions. (c) Schematic representation of an EV showing the functional annotation and localization of enriched proteins in EV extracts across clinical conditions. (d) Biological process enrichment and (e) functional pathway analysis of proteins enriched in EV extracts from HIV patients

Correlation analysis of LFQ protein intensities revealed that the repeatability was higher within (0.823 ± 0.0855) than between (0.694 ± 0.128) clinical context with median Pearson correlation coefficients for EV extracts from breast cancer patients (pooled) of 0.922 ± 0.0478; from ovarian cancer patients of 0.770 ± 0.155; and from HIV patients of 0.778 ± 0.168 (Figure 3a). By contrast, correlation analysis of LPP extracts showed a high median Pearson's r coefficient of 0.902 ± 0.103 across clinical conditions (Figure 3a).

EV‐associated proteins (ANXA2, CD9, EZR, HSPA7, MSN and SDCBP) were detected in EV extracts across all investigated clinical conditions (Figure 3b). EV‐associated proteins were further investigated by functional annotation and gene ontology analyses (Figure 3c, d and e). EV extracts were enriched in EV‐associated proteins (including CD9, SDCBP, FLOT1) and pathways (vesicle‐mediated transport, endocytosis) (Figure 3c, 3d and 3e). In addition, EV extracts were enriched in immune system related proteins (MHC I and II) and pathways (positive regulation and activation of immune responses) across different clinical conditions. None of the 83 identified putative EV contaminants (Table S1) were detected in the EV extracts across clinical conditions.

In conclusion, EV extracts, but not LPP extracts, are characterized by a clinical context‐dependent protein composition while sharing enrichment in immune system related pathways.

### 3.4 Time‐dependent analysis of the proteome landscape of EV and LPP extracts in serial blood plasma samples

Serial blood plasma samples collected from ovarian cancer patients (4 patients, 5 time points) (described in Figure S1), were subjected to sequential SEC and ODG, and EV and LPP extracts were analysed by mass spectrometry‐based proteomics (LC‐MS/MS).

**FIGURE 4:**
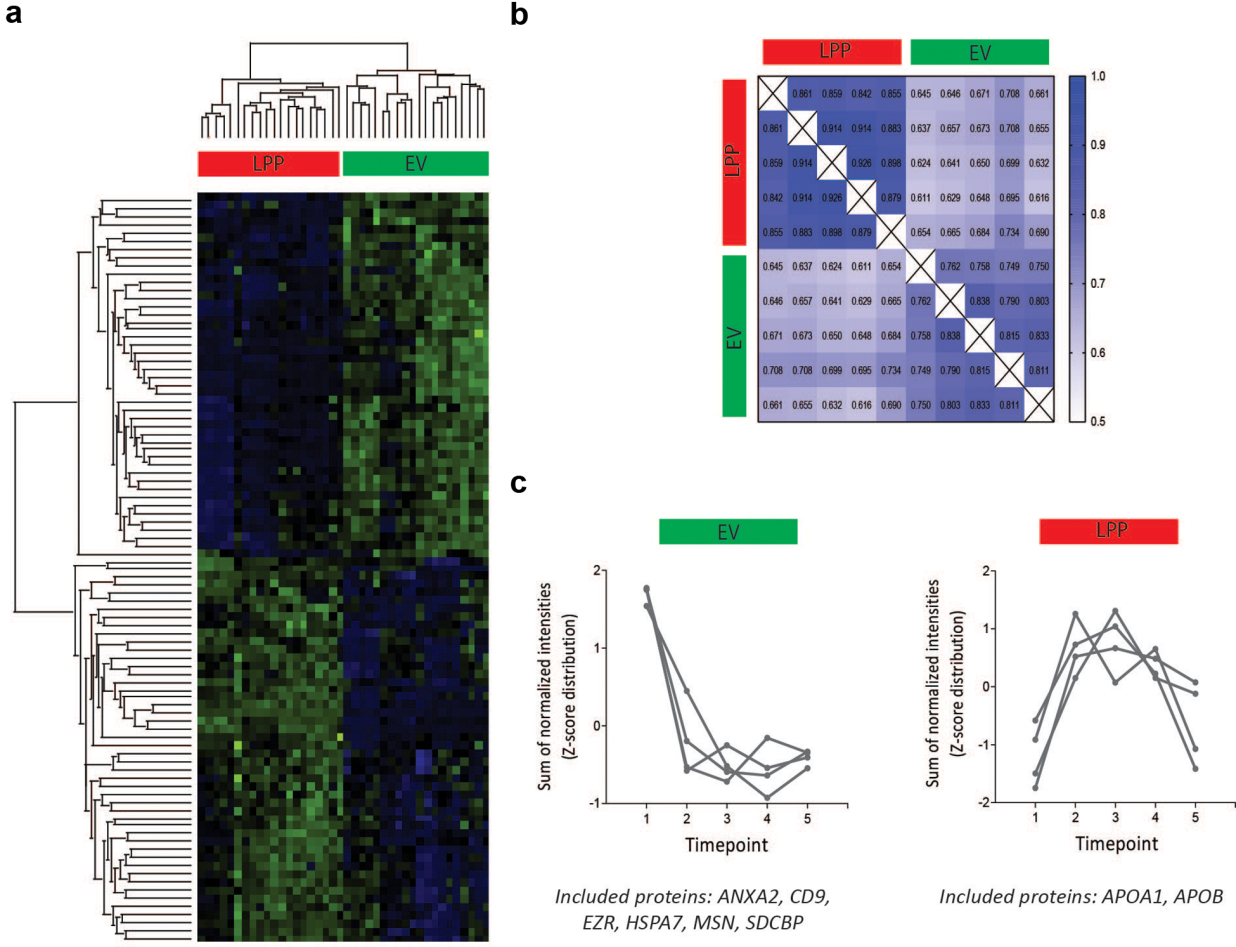
Time‐dependent analysis of the EV and LPP protein landscape using serial blood plasma samples. (a) Unsupervised hierarchical clustering and heatmap analysis of the matched LPP and EV protein composition in serial blood plasma samples (n = 5) from ovarian cancer patients (n = 4). (b) Correlation matrix of the matched LPP and EV protein landscape of one ovarian cancer patient over time. (c) Time‐dependent variation of EV (left) and LPP (right)‐associated proteins in EV and LPP extracts, respectively, by calculating the Z‐scores of the sum of normalized intensities over time

First, unsupervised hierarchical clustering and PCA revealed differential clustering of the LPP and EV extracts across all patients, further confirming the distinct proteome landscape of these two extracts (Figure 4a and Figure S6B). Functional pathway analysis also confirmed depletion of lipoprotein‐ and platelet‐related pathways in all EV extracts (Figure S6A), and none of the 83 identified putative EV contaminants (Table S1) were detected in the EV extracts.

Correlation analyses were performed on the serial samples within each ovarian cancer patient to assess the time‐dependent variation of the proteome landscape in EV and LPP extracts in relation to the earlier evaluated technical variability. As example, the correlation matrix of one patient is depicted in Figure 4b (Figure S6C visualizes a similar analysis for a second patient). The median Pearson correlation coefficient within the EV extracts at different time points (0.797 ± 0.0341) was significantly *P* < 0.0001) lower than within the matched LPP extracts (0.881 ± 0.0289), revealing a time‐dependent variation in the protein composition that was more pronounced in EV extracts compared to LPP extracts. This time‐dependent variation in the protein composition of the EV extract was independent of technical variations, evidenced by the significantly (*P* < 0.0001) higher median Pearson correlation coefficient within EV extracts reported in Figure 2a: 0.877 ± 0.0186. The normalized intensities of the general EV‐associated proteins in EV extracts were decreasing during therapy reflecting the minimal residual disease state (time points 2–5; Figure S1), whereas in the matched LPP extracts lipoprotein marker intensities depended on the fasting state of the patients (time‐point 1 = before surgery, all patients were fasting at the time of blood draw; time‐point 5, two patients were fasting at the time of blood draw) (Figure 4c).

In conclusion, EV extracts are characterized by a time‐dependent variation in protein composition that reflects disease state, while variation in protein composition of LPP extracts reflects fasting state.

### 3.5 Identification and time‐dependent analysis of the small RNA landscape of EV extracts with high repeatability

Similar to the identification of the proteome landscape of EV extracts, first technical replicates (n = 4) derived from a pool of blood plasma collected from breast cancer patients (described in Figure S1), to discard inter‐patient variations, were subjected to sequential SEC and ODG, and total blood plasma and EV extracts were analysed by small RNA sequencing.

**FIGURE 5:**
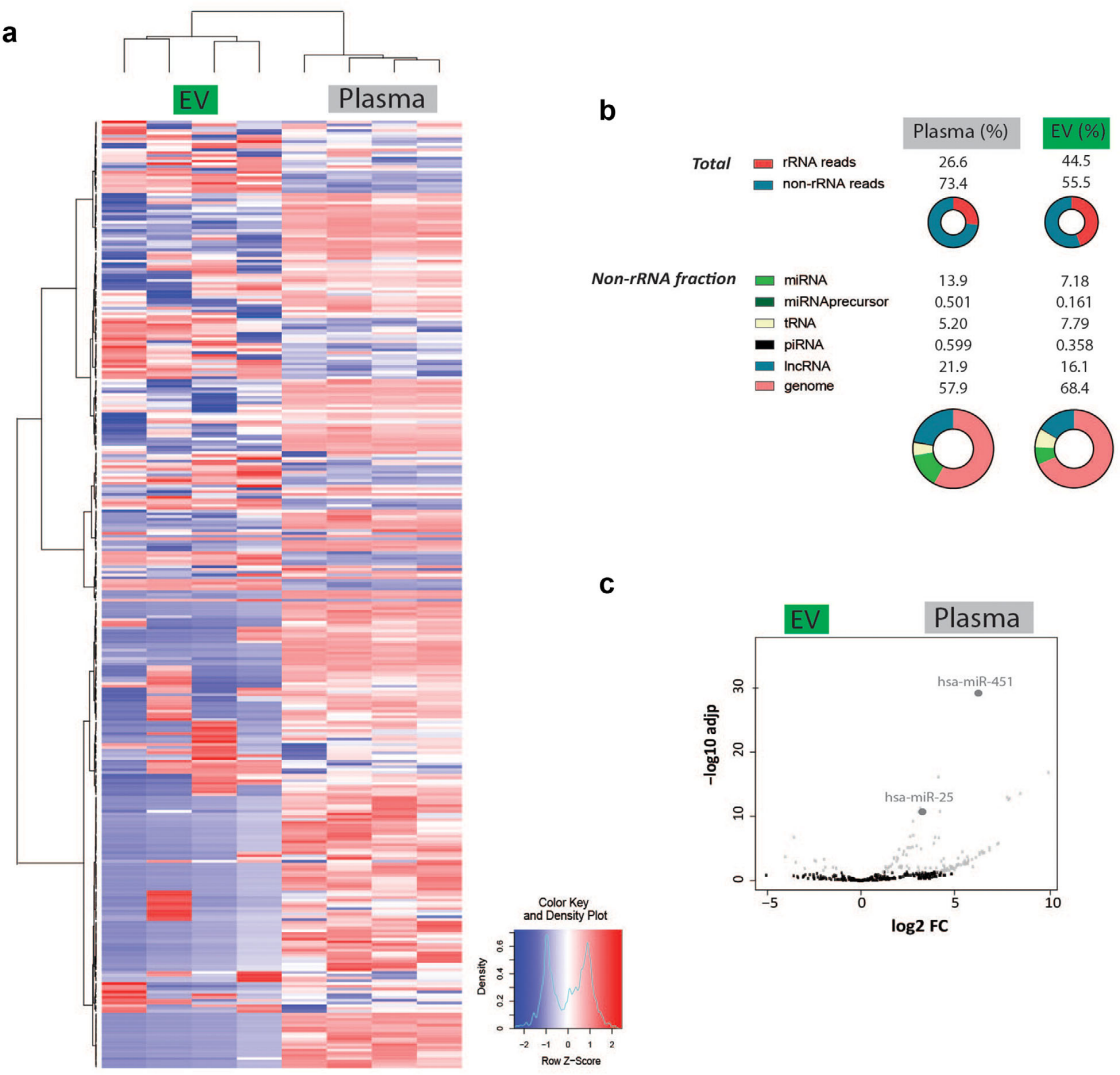
Differential analysis of the small RNA landscape of EV extracts and total blood plasma samples. (a) Unsupervised hierarchical clustering and heatmap analysis. (b) Overview of the different small RNA species in EV extracts and total blood plasma samples. (c) Differential miRNA expression analysis of presumable HDL‐associated (hsa‐miR‐451) and platelet‐associated (hsa‐miR‐25) miRNA in EV extracts and total blood plasma samples.

Unsupervised hierarchical clustering and heatmap analysis revealed a differential transcriptome in EV extracts compared to total blood plasma (Figure 5a). Sequence analysis revealed a diverse collection of small RNA species in EV extracts of which miRNAs accounted for 7.18% of all mappable non‐ribosomal RNA reads (Figure 5b). In line with the proteome landscape of the EV extract, gene set enrichment analysis showed that the miRNA profile of the EV extract was depleted for signatures associated with HDL particles (NES = 1.82, FDR q‐value = 0.017) and platelets (NES = 2.45, FDR q‐value = 0.000) (Figure S7). To further confirm these observations, differential miRNA expression analysis highlighted putative HDL‐associated (hsa‐miR‐451) (Vickers et al., 2011) and platelet‐associated (hsa‐miR‐25) miRNAs (Plé et al., 2012) with a significant higher abundance in total blood plasma (Figure 5c). Other non‐ribosomal RNA fragments in EV extracts included tRNA (7.79%), piRNA (0.358%) and lncRNA (16.1%).

Taken together, these results indicate that the sequential biophysical separation approach depletes LPP‐associated small RNA from EV extracts, and supports the identification of different small RNA species in EV extracts.

To investigate time‐dependent variations in the small RNA landscape of EV extracts unbiased small RNA sequencing was performed on total blood plasma versus EV extracts obtained by sequential SEC and ODG of serial blood plasma samples collected from ovarian cancer patients (4 patients, 5 time points) (described in Figure S1).

**FIGURE 6:**
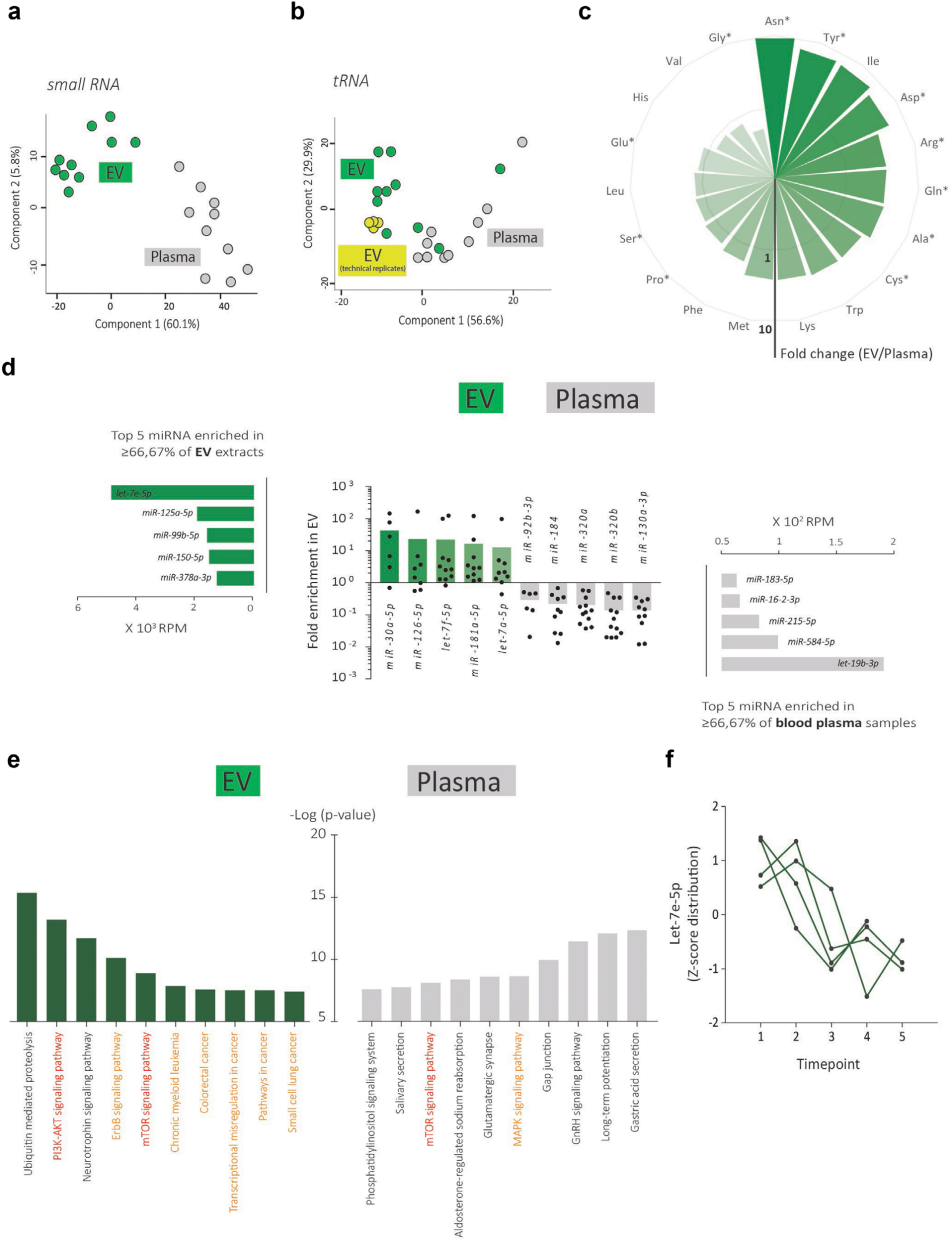
Time‐dependent analysis of the EV small RNA landscape in serial blood plasma samples. (a) PCA of the small RNA composition and (b) tRNA fragment profile of matched EV extracts and total blood plasma samples of ovarian cancer patients (n = 2) over time (n = 5). The technical variability in the breast cancer plasma pool compared to biological variability in the tRNA fragment landscape is indicated in (b). (c) Circular barplot representing the fold change of the relative proportion of pooled tRNA fragments by amino acid codon usage in EV extracts compared to total blood plasma samples. Star indicates codon usage of non‐essential amino acids. Only tRNAs identified in ≥66.67% of all samples are included. (d) MiRNA analysis in matched EV extracts and total blood plasma samples. (e) KEGG pathway enrichment analysis of genes targeted by miRNAs most enriched in EV extracts or miRNAs most enriched in total blood plasma samples. (f) Z‐score distribution of let‐7e‐5p abundance in EV extracts over the collected time points of four ovarian cancer patients

PCA differentially clustered EV extracts from the total blood plasma samples in all ovarian cancer patient samples (Figure 6a). In‐depth sequence analysis revealed time‐dependent variations in the tRNA fragment profile of EV extracts compared to the EV technical replicates of the breast cancer blood plasma pool (Figure 6b). The circular bar plot in Figure 6c represents the fold change of the relative proportion of pooled tRNA fragments by amino acid codon usage in EV extracts compared to total blood plasma samples. Interestingly, 16 out of 19 identified tRNA fragments were relatively more represented in EV extracts compared to total blood plasma samples of which the non‐essential amino acid codon usage was most enriched in EV extracts. In contrast to EV extracts, the tRNA fragment profile in blood plasma was dominated by the Gly family (Figure S8A) explaining the relative enrichment of most other tRNA types in EV.

To further emphasize time‐dependent variations of the small RNA landscape of EV extracts and the advantage of sequential biophysical separation to prepare EV extracts from blood plasma, we compared EV‐enriched miRNAs (green) and total blood plasma enriched miRNAs (grey) over the collected time points of the ovarian cancer patients (Figure 6d). KEGG pathway enrichment analysis of genes targeted by miRNAs was performed resulting in a top 10 of significantly enriched pathways including . cancer‐related pathways (orange) specifically in EV extracts (Figure 6e). Particularly, the PI3K‐AKT and mTOR signalling pathways (red), frequently altered in high grade serous ovarian carcinoma (Bell et al., 2011) showed significant enrichment in EV extracts. Interestingly, let‐7e‐5p, a regulator of the two signalling pathways (and also a highly abundant miRNA enriched in the EV extract, see Figure 6d) substantially decreased during therapy (Figure 6f, 4 ovarian cancer patients, 5 time points). All data strongly indicate that a therapy‐induced effect on the small RNA landscape can be identified in EV extracts from the systemic circulation. In addition, let‐7e‐5p levels significantly correlated to the earlier reported reduction in general EV‐associated protein intensities with treatment time (Figure 4c and Figure S8B).

## 4 DISCUSSION

Assessment of biomolecular landscape of EV in blood plasma for biomarker development requires their preparation with high repeatability. Hereto, we optimized sequential size and density‐based fractionation from blood plasma, performed proteomics and small RNA sequencing to demonstrate the technical repeatability of the fractionation strategy, and studied the differential proteome and transcriptome of EV extracts to draft meaningful conclusions on clinical context‐dependent and time‐dependent variations in their biomolecular landscape.

Size‐exclusion chromatography prepares crude extracts from blood plasma retaining 1.4%, 9.67% and 73.0% of APOA1‐containing LPP, APOB‐containing LPP and CD9‐containing EV, respectively, and a minority of soluble proteins (1.1% of total plasma protein). However, since LPP have an estimated 6 orders of magnitude higher concentration than EV, LPP still substantially outnumber EV in crude extracts as confirmed by TEM and proteomics. Density gradient centrifugation fractionates crude extracts, obtained by SEC, into LPP and EV extracts. Indeed, LPP extracts retained 85.2% and 95.4% of APOA1‐containing LPP and APOB‐containing LPP, respectively. In accordance, mass spectrometry identified a 5 to 7‐fold higher percentage of HDL‐associated and LDL‐associated proteins in LPP extracts compared to EV extracts, and TEM revealed LPP extracts depleted of EV and vice versa. Although SEC is often implemented as a stand‐alone method we demonstrated that the proteome of crude extracts closely resembles the proteome of LPP extracts, while a differential proteome is identified in EV extracts (Figure 2a and d). Variations to our fractionation strategy have been reported, but the unbiased assessment of the performance of blood plasma fractionation in terms of methodological repeatability, although crucial to assess the potential of EV as biomarkers, was not addressed (Karimi et al., 2018; Onódi et al., 2018; Zhang et al., 2020). Proteomics and small RNA sequencing of technical replicates revealed high methodological repeatability (Figure 2a, b and d and Figure 5a). The use of robot‐assisted gradient preparation and fraction collection together with reference materials such as trackable recombinant EV (rEV) may further ensure the reproducibility of sequential SEC and ODG (Geeurickx et al., 2019, 2021; Tulkens et al., 2020a).

Previously we reported EV recoveries of 30% for sequential size and density‐based fractionation of blood plasma (Geeurickx et al., 2019). Hereto, it is important to consider that we study a selection of EV and LPP. In this manuscript we select LPP and EV for size (LPP and EV particles: SEC fractions 5 and 6, LPP and EV particles: 50–250 nm) and density (LPP particles: 1.04‐1.07 g/ml; EV particles: 1.09–1.10 g/ml). To further scrutinize the biomarker potential and biological role of different LPP species and EV subtypes, LPP and EV eluting in SEC fractions (Allen et al., 2018; Burillo et al., 2016; Coumans et al., 2017; Dashty et al., 2014; Emmens et al., 2018; Geeurickx et al., 2019; Geyer et al., 2017; Hendrix, 2021; Johnsen et al., 2019; Shah et al., 2013) can be further processed by a similar density gradient centrifugation strategy. Alternatively, asymmetric flow field flow fractionation (AF4) is highly valuable and can be used to further analyse LPP and EV in crude, LPP and EV extracts. Indeed, AF4 fractionation of LPP from blood serum has been reported to provide quantitative information on LPP subtypes and their composition (Kuklenyik et al., 2018); and AF4 fractionation of EV from cell culture supernatant was implemented to provide quantitative information on small and large EV (Zhang & Lyden, 2019; Zhang et al., 2018). Besides LPP and EV, exomeres, a type of small (< 50 nm), non‐membranous nanoparticle that contains lipids, may constitute a third type of lipid‐carrying particle in blood plasma (Zhang & Lyden, 2019).

A group of proteins, identified by mass spectrometry‐based proteomics, was significantly enriched (Student's t‐test corrected for multiple testing, *P* < 0.05) in LPP extracts compared to EV extracts. We implemented this knowledge to develop an extended list of putative non‐EV associated proteins in blood plasma (Table S1) (Théry et al., 2018). This list is composed of proteins that are retained by SEC in the crude extract and omits soluble proteins that elute in later SEC fractions and thus do not have the propensity to aggregate or adhere to lipid‐carrying particles. Interestingly, functional pathway analysis of these listed proteins revealed the enrichment of non‐EV associated pathways, such as CM remodelling, blood coagulation and fibrin clot formation, and complement activation. Of note, across clinical conditions, none of the putative non‐EV associated proteins were identified in EV extracts.

Similar to the identification of putative non‐EV associated proteins in blood plasma, differential analysis of blood plasma fractions may be instrumental to increase our understanding of the protein corona at the EV surface. Taking into account the three following criteria, putative EV corona proteins may be identified: (1) proteins have a similar retention time during SEC and thus are identified in the crude extract, (2) proteins have a similar density as EV and thus are significantly enriched (Student's t‐test corrected for multiple testing, *P* < 0.05) in EV extracts (relative to LPP extracts); and (3) proteins are annotated as secreted proteins, yet consistently identified in EV extracts (Figure S9A). Some of these proteins are known to interact with transmembrane receptors identified on EV (such as F8/VWF, that interacts with the ITGA2B/ITGB3 complex), and as has been described for nanomaterials (Whitwell et al., 2016) and viruses (Ezzat et al., 2019), the final amount of protein incorporation in the EV corona seems to correlate (Spearman's ρ = 0.456, *P* = 0.033) with blood plasma concentration (Figure S9B). Further investigations are required to confirm these putative EV corona proteins, including but not limited to studying whether or not highly abundant soluble apolipoproteins in the bloodstream, that have been shown to dominate the protein corona of nanomaterials (Pattipeiluhu et al., 2020), adhere to the EV surface and whether sequential size and density‐based fractionation of blood plasma only captures the hard EV protein corona (i.e., proteins directly adsorbed to the EV surface) or whether a potentially important soft EV protein corona dissociates during fractionation. Of note, A2M and LGALS3BP, two putative EV corona proteins, have been reported as EV‐associated biomarkers (Hoshino et al., 2020).

Analysis of the small RNA landscape of EV extracts by RNA sequencing revealed non‐ribosomal RNA reads such as miRNA, tRNA and lncRNA (Figure 5b). A substantial proportion of this landscape was not detectable in total blood plasma. The starting volume of total blood plasma (200 μl) was not identical to the starting volume of total blood plasma (3 ml) used to prepare EV extracts, which may contribute to the differences in RNA abundance between EV extracts and total blood plasma. However, putative miRNA signatures of HDL particles (Vickers et al., 2011) were significantly depleted in EV extracts (Figure S7).

While there is growing evidence suggesting that EV reflect biological functions, the clinical translation of EV as biomarkers is thus far limited (De Wever & Hendrix, 2019). We identify clinical context‐dependent but also time‐dependent variations in the protein landscape of EV extracts. The inclusion of blood plasma samples of fasting versus non‐fasting donors further highlights that the biomolecular landscape of EV extracts is not dependent on the fasting state. In accordance, we also reveal time‐dependent variations in the small RNA landscape of EV extracts. Let‐7e‐5p, miR‐125a‐5p, miR‐99b‐5p, miR‐150‐5p, miR‐378a‐3p were the most abundant enriched miRNAs in EV extracts compared to total blood plasma and associated with cancer related pathways including PI3K‐AKT and mTOR (Figure 6d and e). Moreover, EV‐associated miRNA let‐7e‐5p substantially decreased during the treatment course of ovarian cancer patients. tRNAs, a major class of noncoding RNA, deliver amino acids to the ribosome during protein synthesis that support cancer cell growth and proliferation. Furthermore, tRNAs function in the regulation of gene expression and epigenetics, particularly in the context of immunomodulation (Chiou et al., 2018) and cancer progression (Goodarzi et al., 2016). tRNA fragments are generated by ribonucleases including Dicer and angiogenin (Li et al., 2018) and are preferentially enriched in EV (Chiou et al., 2018). Similar to other research focusing on blood‐derived EV (Amorim et al., 2017), we found that tRNA (presumably fragmented tRNA) were well represented in the non‐ribosomal set of sequences originating from EV extracts. We identified miRNA and tRNA equally abundant in EV extracts from blood plasma (Figure 5b). Moreover, we showed that compared to total blood plasma, EV carry a disparate tRNA codon landscape enriched in codon usage of non‐essential amino acids and that this tRNA codon landscape shows time‐dependent variations during cancer treatment (Figure 6b and c).

In conclusion, to enable the comprehensive investigation of clinical context‐dependent and time‐dependent variations in the biomolecular landscape of EV in blood plasma we evaluated and implemented sequential size and density‐based fractionation. We demonstrated the technical repeatability of the fractionation approach. Variations in the biomolecular landscape of EV extracts but not LPP extracts, inferred disease condition (breast cancer, ovarian cancer and HIV) and treatment response (ovarian cancer). Finally, differential analysis of crude, EV and LPP extracts allowed us to extend the list of putative non‐EV associated proteins and we discuss how a similar strategy may assist the identification of the EV protein corona.

## COMPETING INTERESTS

The authors declare that they have no competing interests.

## AUTHOR CONTRIBUTIONS

Glenn Vergauwen, Joeri Tulkens and Cláudio Pinheiro planned, designed and performed the experiments. Glenn Vergauwen, Joeri Tulkens and Cláudio Pinheiro performed blood plasma fractionation and characterization. Glenn Vergauwen, Joeri Tulkens, Cláudio Pinheiro, Sándor Dedeyne, Kris Gevaert and Francis Impens performed and analysed proteomics experiments. Glenn Vergauwen, Joeri Tulkens, Cláudio Pinheiro, Francisco Avila Cobos, Pieter Mestdagh and Jo Vandesompele performed and analysed RNA seq experiments. Ilkka Miinalainen performed electron microscopy. Marie‐Angélique De Scheerder, Linos Vandekerckhove, Geert Braems and Hannelore Denys collected patient material. Hannelore Denys, Olivier De Wever and An Hendrix initiated, designed and conceptualized the study. Joeri Tulkens and An Hendrix wrote the manuscript. All authors revised the manuscript for important intellectual content and approved the final version of the manuscript.

## Supporting information

**Fig. S1. Schematic overview of the blood samples used in this study with description of clinical parameters**. (**A**) Overview of the use of different clinical samples in the study. (**B**) Baseline clinical characteristics of the breast cancer and HIV patients included in the study. (**C**) Baseline clinical characteristics of the ovarian cancer patients included in the study

Fig. S2. Additional characterization of crude, LPP and EV extracts obtained by sequential biophysical fractionation of blood plasma samples. After size‐exclusion chromatography (SEC) starting from 2 ml healthy donor blood plasma, all consecutive SEC fractions were analysed by (A) Coomassie Brilliant Blue staining (equal volumes loaded), (B) protein concentration measurement and (C) nanoparticle tracking analysis. (D) Particle enrichment factor analysis relative to proteins, APOA1‐containing and APOB‐containing LPP in total blood plasma versus crude and EV extracts. (E) Western blot analysis for EV (FLOT1) and APOA1‐containing LPP of consecutive SEC fractions obtained using a commercial SEC column. (F) Western blot analysis of EV (FLOT1) and APOA1‐containing LPP of consecutive fractions obtained after SEC with 0.5 ml healthy donor blood plasma. All SEC fractions were loaded in equal volumes for western blot analysis and Coomassie brilliant blue staining.

**Fig. S3. Western blot analysis of crude extract (SEC 5–6) and EV extract (1.09‐1.10** **g/ml)**. Samples were obtained by spiking 10e10 GFP‐positive EV in PBS followed by size‐exclusion chromatography and OptiPrep density gradient centrifugation.

Fig. S4. Kernel density plots representing the distribution of protein differences between matched crude, LPP and EV extracts. Kernel density plots representing the distribution of protein differences between matched (A) crude and EV extracts, (B) crude and LPP extracts and (C) EV and LPP extracts.

**Fig. S5. Additional characterization of the proteome landscape of crude, LPP and EV extracts**. (**A**) Relative LFQ intensities for EV‐associated proteins (CD9 and ANXA2), lipoproteins (APOA1 and APOB) and other contaminants (F2 and C3) in the different extracts. (**B**) Graphical representation of the HDL (left) and LDL (right) association of proteins enriched in LPP, crude and EV extracts. (**C**) Functional pathway analysis of EV and crude extract protein landscapes.

**Fig. S6. Additional characterization of time‐dependent variations in the protein landscape of LPP and EV extracts**. (**A**) Functional pathway analysis and (**B**) PCA of EV and LPP extract protein landscapes of ovarian cancer patients (n = 4) over the serial time points (n = 5). (**C**) Correlation matrix of the matched LPP and EV protein landscapes of one ovarian cancer patient.

**Fig. S7. Gene Set Enrichment Analyses for HDL‐ and platelet‐associated miRNAs in EV extracts**. (**A**) Gene Set Enrichment Analysis for HDL‐associated miRNAs (top 50) (Vickers et al., 2011) in EV extracts. (**B**) Gene Set Enrichment Analysis for platelet‐associated miRNAs (top 50) (Plé et al., 2012) in EV extracts.

**Fig. S8. Additional characterization of the dynamic small RNA landscape of EX extracts and total blood plasma samples**. (**A**) Percentage of tRNA^Gly^ in EV extracts and total blood plasma samples based on the total number of sample reads assigned to tRNAs (Mann‐Whitney U test, *P* = 0.0029). (**B**) Spearman correlation analysis between the Z‐score distributions of let‐7e‐5p and normalized EV‐associated protein intensities over the different collected time points.

**Fig. S9. Characterization of the protein corona at the EV surface**. (**A**) Graphical representation of the selected putative corona proteins (and their functional annotation) at the EV surface. (**B**) Spearman correlation analysis of LFQ intensities EV corona proteins with blood plasma concentration.

**Table S1. Overview of the 83 selected putative non‐EV associated proteins**. Overview of the 83 selected putative non‐EV associated proteins ranked on p‐values (Student's t‐test corrected for multiple testing, *P* < 0.05) representing the chance to be absent in EV extracts. Proteins in red were never detected across all analysed EV extracts in this study.

Supplementary information

## Data Availability

All data needed to evaluate the conclusions in the paper are present in the paper and/or the Supplementary Materials. Additional data related to this paper may be requested from the authors.
