# Supplementary figures and images for: Robust sequential biophysical fractionation of blood plasma to study variations in the biomolecular landscape of systemically circulating extracellular vesicles across clinical conditions
Source: J Extracell Vesicles. 2021 Aug 14;10(10):e12122. doi: 10.1002/jev2.12122 (PMC8363909; doi:10.1002/jev2.12122)

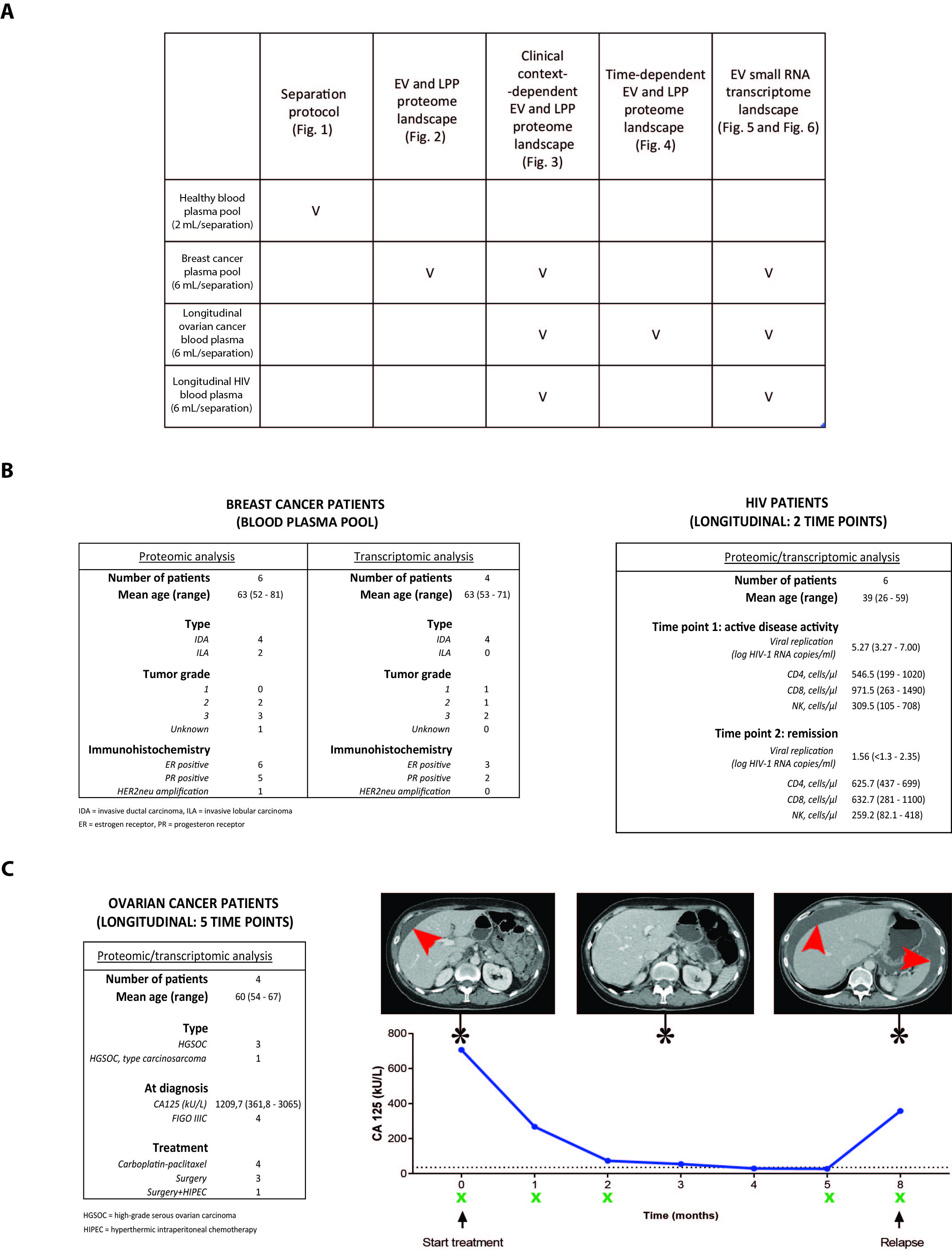

Supplement: Supplementary file 1 — Fig. S1. Schematic overview of the blood samples used in this study with description of clinical parameters. (A) Overview of the use of different clinical samples in the study. (B) Baseline clinical characteristics of the breast cancer and HIV patients included in the study. (C) Baseline clinical characteristics of the ovarian cancer patients included in the study [file JEV2-10-e12122-s002.jpg]

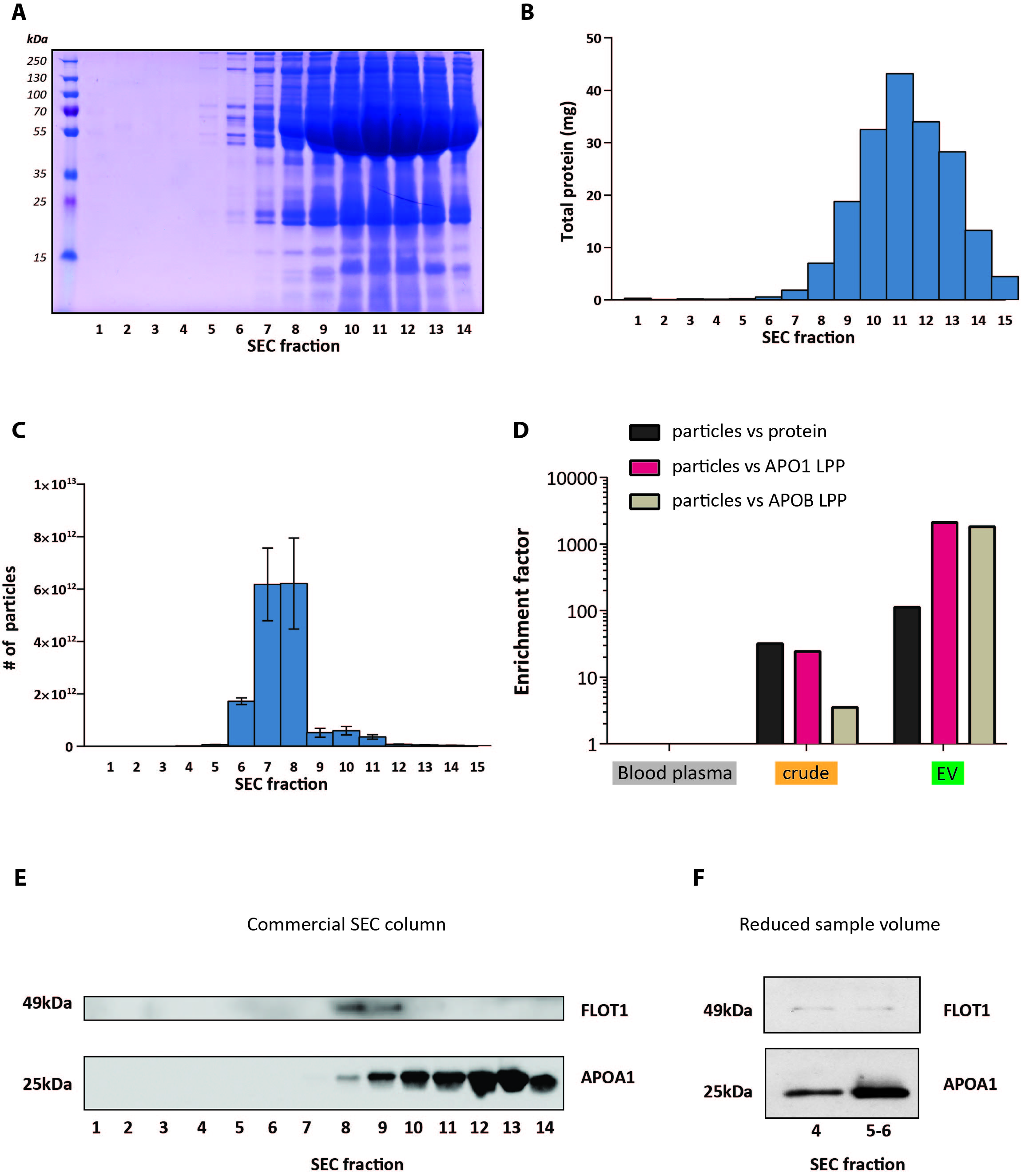

Supplement: Supplementary file 2 — Fig. S2. Additional characterization of crude, LPP and EV extracts obtained by sequential biophysical fractionation of blood plasma samples. After size‐exclusion chromatography (SEC) starting from 2 ml healthy donor blood plasma, all consecutive SEC fractions were analysed by (A) Coomassie Brilliant Blue staining (equal volumes loaded), (B) protein concentration measurement and (C) nanoparticle tracking analysis. (D) Particle enrichment factor analysis relative to proteins, APOA1‐containing and APOB‐containing LPP in total blood plasma versus crude and EV extracts. (E) Western blot analysis for EV (FLOT1) and APOA1‐containing LPP of consecutive SEC fractions obtained using a commercial SEC column. (F) Western blot analysis of EV (FLOT1) and APOA1‐containing LPP of consecutive fractions obtained after SEC with 0.5 ml healthy donor blood plasma. All SEC fractions were loaded in equal volumes for western blot analysis and Coomassie brilliant blue staining. [file JEV2-10-e12122-s011.jpg]

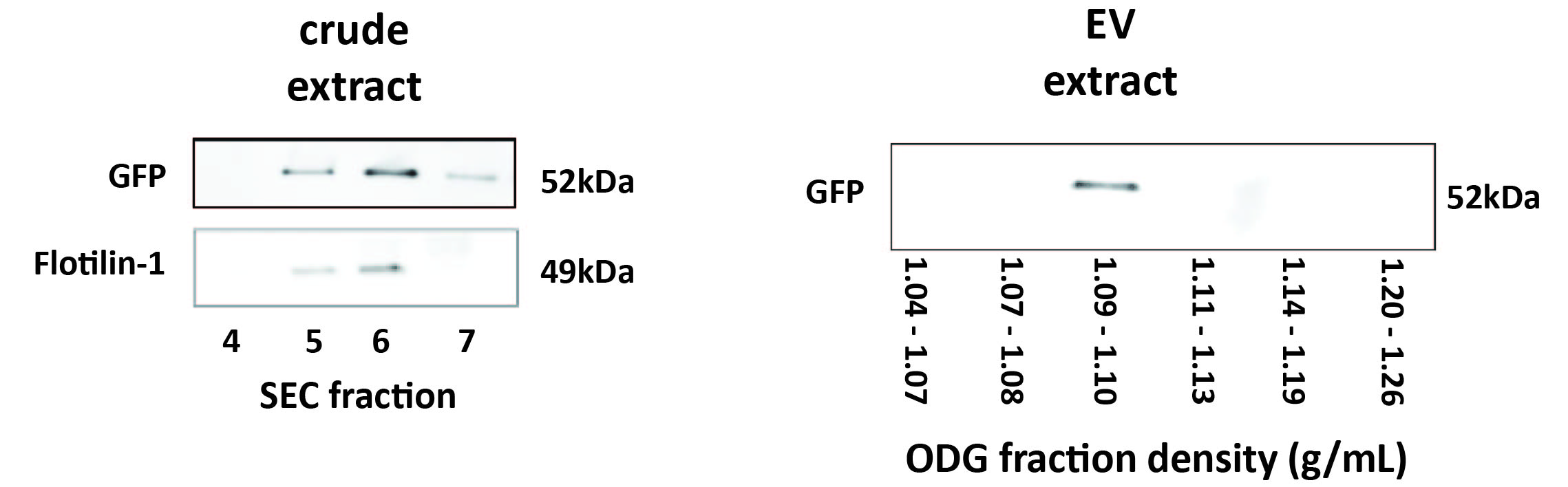

Supplement: Supplementary file 3 — Fig. S3. Western blot analysis of crude extract (SEC 5–6) and EV extract (1.09‐1.10 g/ml). Samples were obtained by spiking 10e10 GFP‐positive EV in PBS followed by size‐exclusion chromatography and OptiPrep density gradient centrifugation. [file JEV2-10-e12122-s005.jpg]

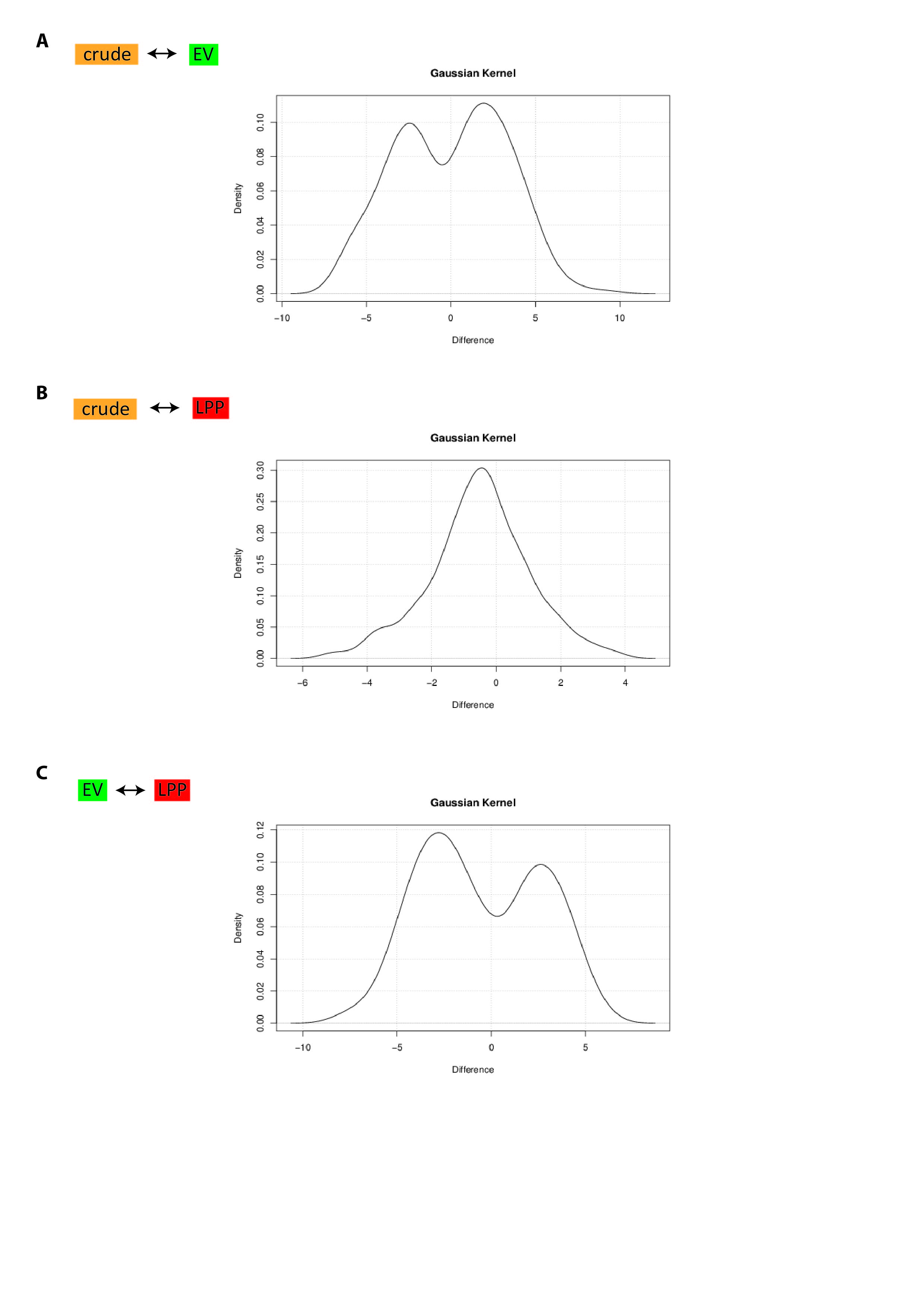

Supplement: Supplementary file 4 — Fig. S4. Kernel density plots representing the distribution of protein differences between matched crude, LPP and EV extracts. Kernel density plots representing the distribution of protein differences between matched (A) crude and EV extracts, (B) crude and LPP extracts and (C) EV and LPP extracts. [file JEV2-10-e12122-s008.jpg]

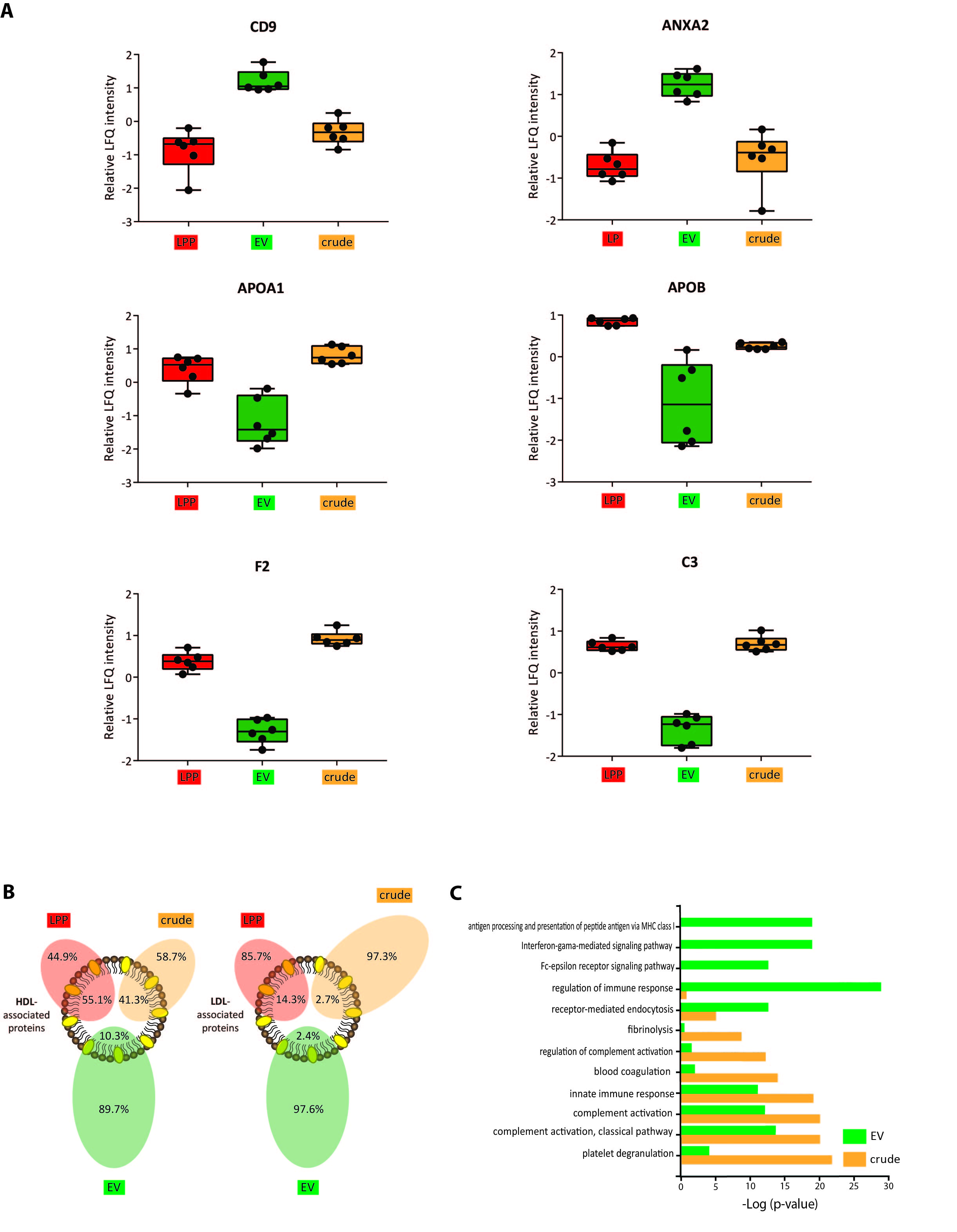

Supplement: Supplementary file 5 — Fig. S5. Additional characterization of the proteome landscape of crude, LPP and EV extracts. (A) Relative LFQ intensities for EV‐associated proteins (CD9 and ANXA2), lipoproteins (APOA1 and APOB) and other contaminants (F2 and C3) in the different extracts. (B) Graphical representation of the HDL (left) and LDL (right) association of proteins enriched in LPP, crude and EV extracts. (C) Functional pathway analysis of EV and crude extract protein landscapes. [file JEV2-10-e12122-s004.jpg]

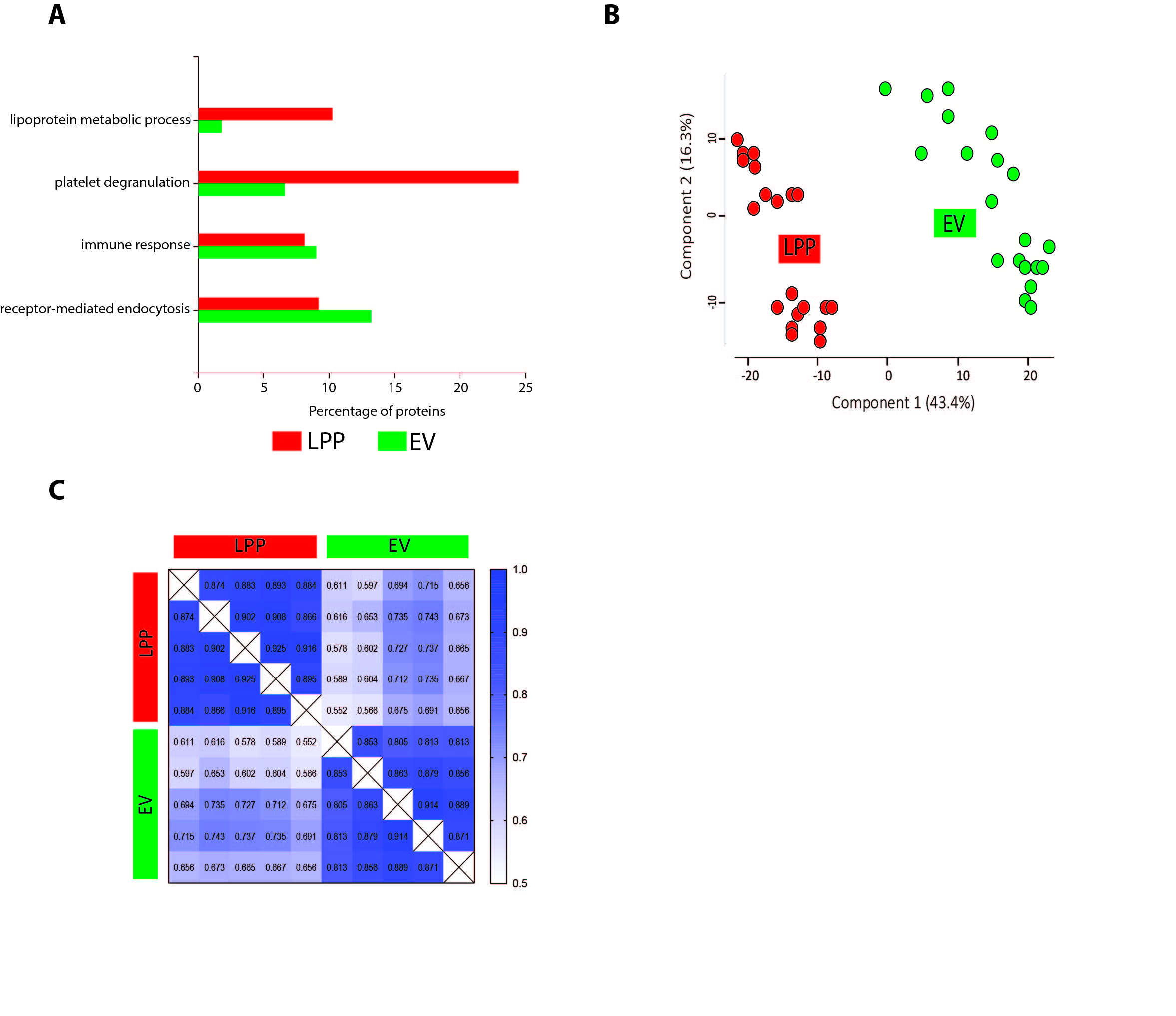

Supplement: Supplementary file 6 — Fig. S6. Additional characterization of time‐dependent variations in the protein landscape of LPP and EV extracts. (A) Functional pathway analysis and (B) PCA of EV and LPP extract protein landscapes of ovarian cancer patients (n = 4) over the serial time points (n = 5). (C) Correlation matrix of the matched LPP and EV protein landscapes of one ovarian cancer patient. [file JEV2-10-e12122-s001.jpg]

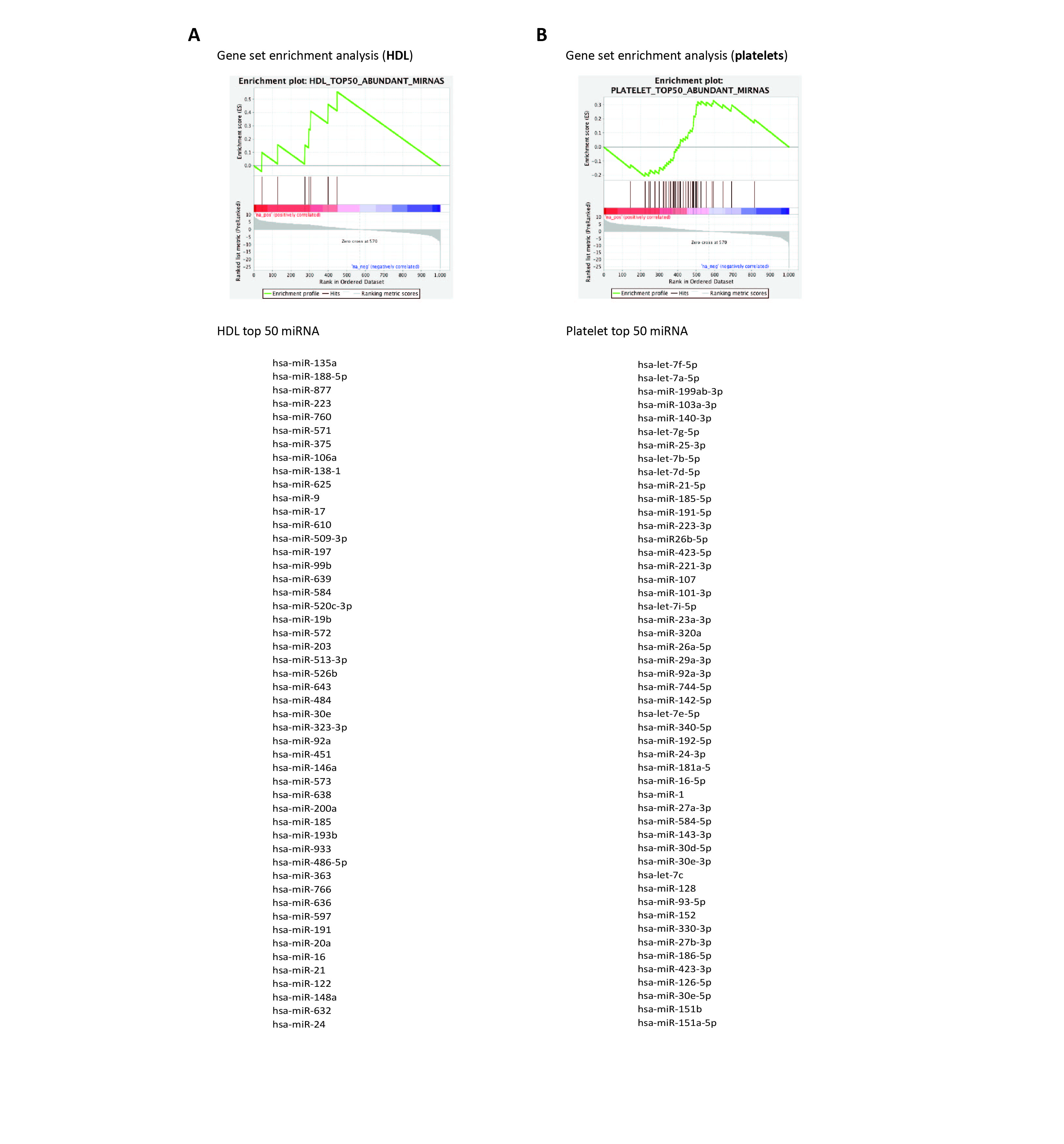

Supplement: Supplementary file 7 — Fig. S7. Gene Set Enrichment Analyses for HDL‐ and platelet‐associated miRNAs in EV extracts. (A) Gene Set Enrichment Analysis for HDL‐associated miRNAs (top 50) (Vickers et al., 2011) in EV extracts. (B) Gene Set Enrichment Analysis for platelet‐associated miRNAs (top 50) (Plé et al., 2012) in EV extracts. [file JEV2-10-e12122-s009.jpg]

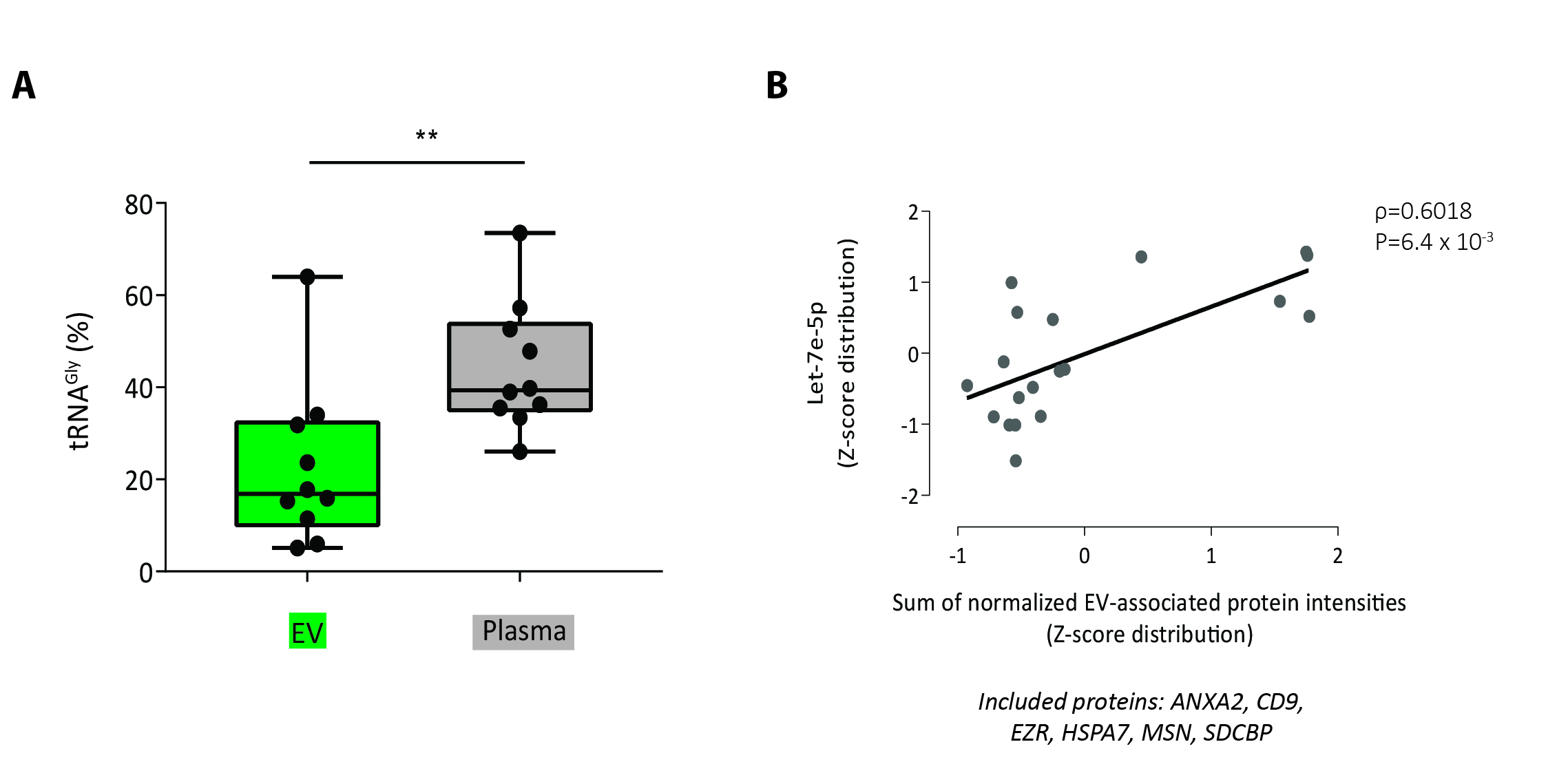

Supplement: Supplementary file 8 — Fig. S8. Additional characterization of the dynamic small RNA landscape of EX extracts and total blood plasma samples. (A) Percentage of tRNAGly in EV extracts and total blood plasma samples based on the total number of sample reads assigned to tRNAs (Mann‐Whitney U test, P = 0.0029). (B) Spearman correlation analysis between the Z‐score distributions of let‐7e‐5p and normalized EV‐associated protein intensities over the different collected time points. [file JEV2-10-e12122-s006.jpg]

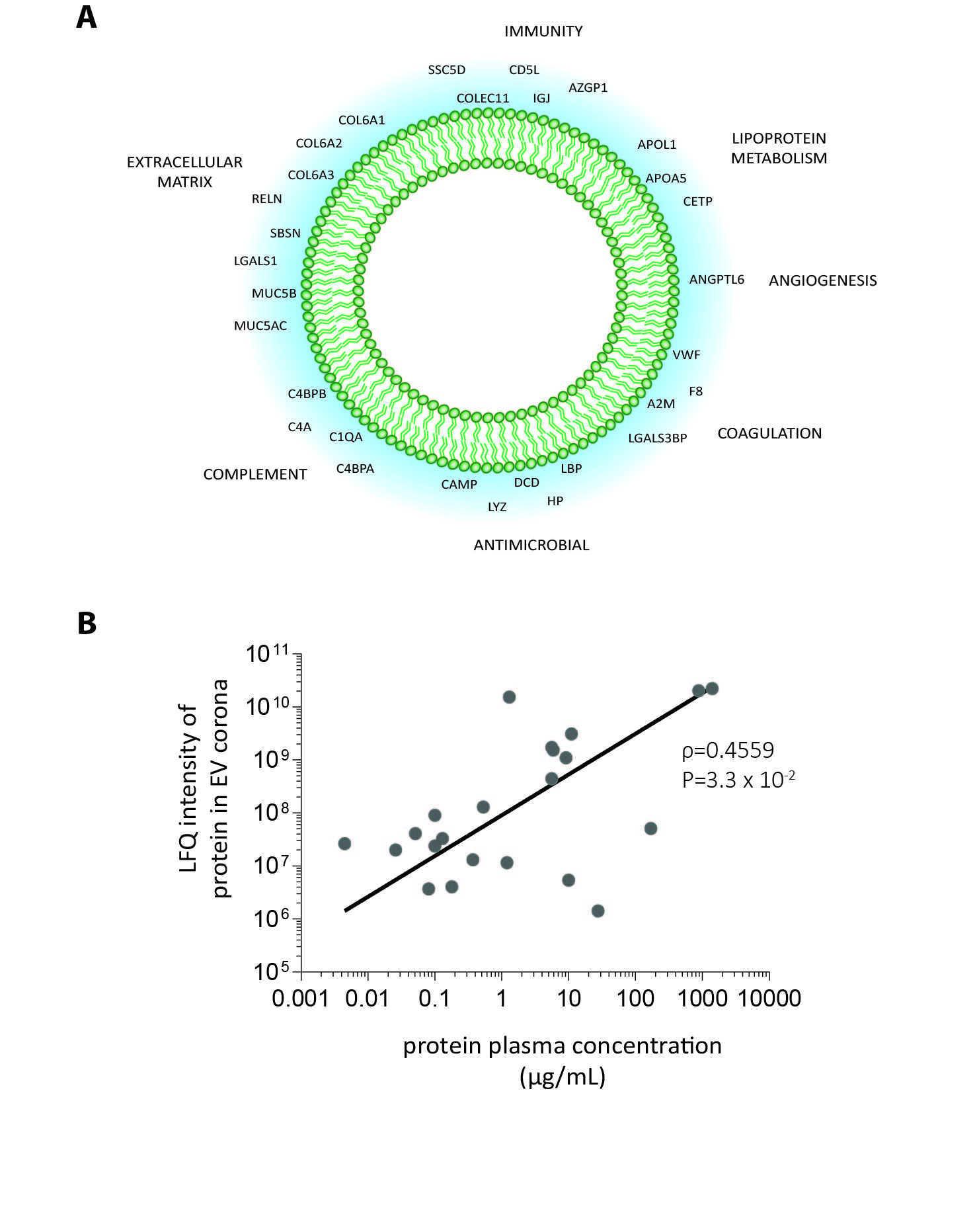

Supplement: Supplementary file 9 — Fig. S9. Characterization of the protein corona at the EV surface. (A) Graphical representation of the selected putative corona proteins (and their functional annotation) at the EV surface. (B) Spearman correlation analysis of LFQ intensities EV corona proteins with blood plasma concentration. [file JEV2-10-e12122-s003.jpg]

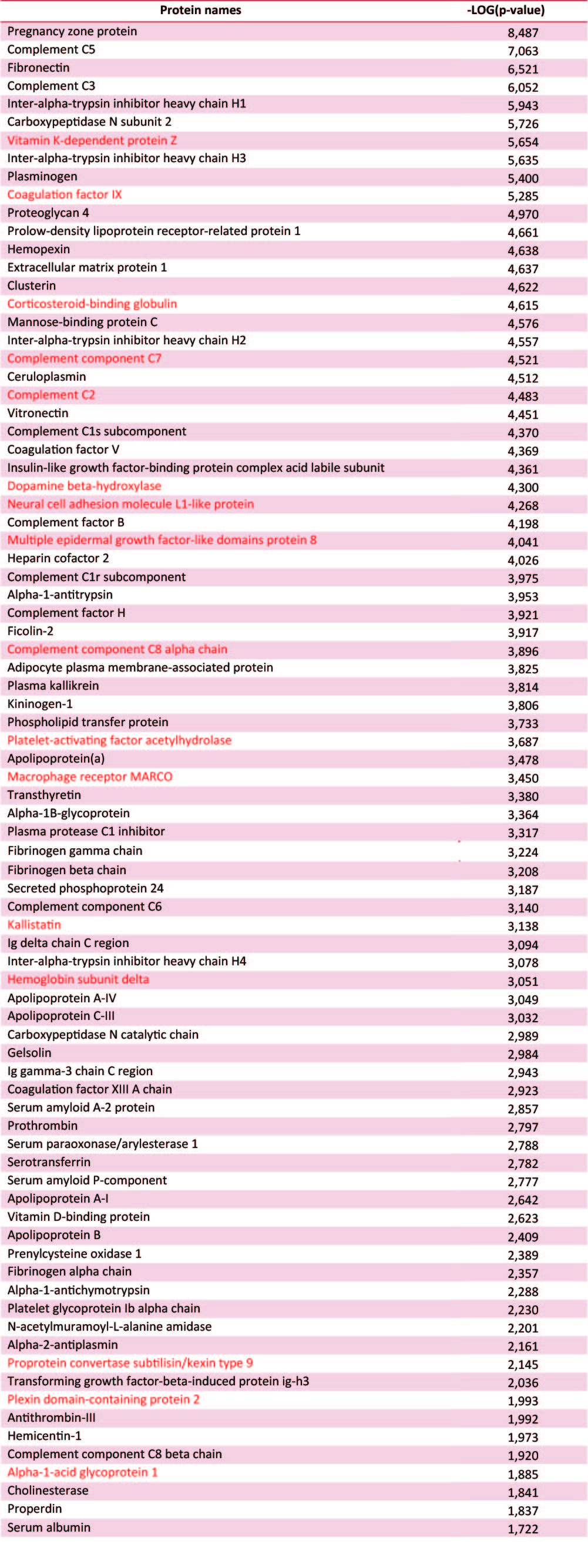

Supplement: Supplementary file 10 — Table S1. Overview of the 83 selected putative non‐EV associated proteins. Overview of the 83 selected putative non‐EV associated proteins ranked on p‐values (Student's t‐test corrected for multiple testing, P < 0.05) representing the chance to be absent in EV extracts. Proteins in red were never detected across all analysed EV extracts in this study. [file JEV2-10-e12122-s010.jpg]
