# Supplementary material for: Robust sequential biophysical fractionation of blood plasma to study variations in the biomolecular landscape of systemically circulating extracellular vesicles across clinical conditions
Source: J Extracell Vesicles. 2021 Aug 14;10(10):e12122. doi: 10.1002/jev2.12122 (PMC8363909; doi:10.1002/jev2.12122)
Supplement: Supplementary file 11 — Supplementary information [file JEV2-10-e12122-s007.docx]

**Supplementary Material and Methods**

**Generation and separation of GFP-positive EV**

***Cell culture***

The MCF-7 cell line (ATCC, Manassas, VA, USA) was stably transfected with peGFP-C1 vector (Clontech, Mountain View, California, USA) containing the GFP-Rab27b fusion protein, as previously described (MCF-7 GFP-Rab27b) (Hendrix et al, JNCI 2010, PMID: 20484105). MCF-7 GFP-Rab27b cells were cultured in Dulbecco’s Modified Eagle Medium supplemented (DMEM) with 10% fetal bovine serum, 100 U/mL penicillin, 100 µg/mL streptomycin and 1 mg/mL G418. Presence of Mycoplasma contamination was routinely tested using MycoAlert Mycoplasma Detection Kit (Lonza, Verviers, Belgium).

***Preparation of conditioned medium***

To prepare conditioned medium, 4 x 10^8^ MCF-7 Rab27b-GFP cells were washed once with DMEM, followed by two washing steps with DMEM supplemented with 0.5% EV-depleted fetal bovine serum (EDS). EDS was obtained after 18h ultracentrifugation at 100,000g and 4°C (SW55 Ti rotor, Beckman Coulter, Fullerton, California, USA), followed by 0.22µm filtration. Flasks were incubated at 37°C and 10% CO_2_ with 15 mL DMEM containing 0.5% EDS. After 24h conditioned medium was collected and centrifuged for 10 min at 200g and 4°C. Cell counting was performed with trypan blue staining to assess cell viability (Cell Counter, Life Technologies, Carlsbad, California, USA). The supernatant was passed through a 0.45µm cellulose acetate filter (Corning, New York, USA) and conditioned medium was concentrated at 4°C approximately 250 times using a 10 kDa Centricon Plus-70 centrifugal unit (Merck Millipore, Billerica, Massachusetts, USA). After filtering through a 0.22µm filter (Whatman, Dassel, Germany), concentrated conditioned medium was used for Optiprep density gradient centrifugation.

***Separation of GFP-positive EV***

OptiPrep (Axis-Shield, Oslo, Norway) density gradients were prepared as previously described^27^. Briefly, a discontinuous iodixanol gradient was prepared by layering 4 mL of 40%, 4 mL of 20%, 4 mL of 10% and 3.5 mL of 5% iodixanol in a 16.8 mL open top polyallomer tube (Beckman Coulter, Fullerton, California, USA). One milliliter of concentrated conditioned medium was placed on top of the gradient, followed by 18h ultracentrifugation at 100,000g and 4°C using SW 32.1 Ti rotor (Beckman Coulter, Fullerton, California, USA). Fractions of 1 mL were collected and fraction 8 and 9 were pooled, corresponding to a density of 1.09-1.10 g/mL. Pooled fractions were diluted to 15 mL with phosphate-buffered saline (PBS), followed by 3h ultracentrifugation at 100,000g and 4°C using SW 32.1 Ti rotor (Beckman Coulter, Fullerton, California, USA). Resulting pellets were resuspended in 100 µL PBS and stored at -80°C until further use. Cell culture derived EV from MCF-7 Rab27b-GFP cells were previously characterized and are referred hereafter as GFP-positive EVs (23,24)

***Antibodies***

Anti-green fluorescent protein (GFP) (1:1000, MAB3580, Chemicon, Temecula, California, USA).

**GFP-spike in control experiment**

Healthy donor plasma (2 mL) was spiked with 10^10^ GFP-positive EVs, followed by size-exclusion chromatography and OptiPrep density gradient centrifugation as described in the material and methods section.”
